# An in vitro-based hazard assessment of liquid smoke food flavourings

**DOI:** 10.1007/s00204-021-03190-1

**Published:** 2021-11-20

**Authors:** Erica Selin, Geeta Mandava, Alexandra-Livia Vilcu, Agneta Oskarsson, Johan Lundqvist

**Affiliations:** Department of Biomedical Science and Veterinary Public Health, Swedish University of Agricultural Sciences, Box 7028, 750 07 Uppsala, Sweden

**Keywords:** Smoke flavouring, Commercial liquid smoke flavouring, Food additives, Bioassays, Bioanalytical tool, Effect-based methods

## Abstract

Liquid smoke products are widely used as a food additive to create a desired smoke flavour. These products may contain hazardous chemicals generated during the wood-burning process. However, the toxic effects of these types of hazardous chemicals constituting in the commercially available products are largely unknown. Therefore, a test battery of cell-based in vitro methods, covering different modes of actions of high relevance to human health, was applied to study liquid smoke products. Ten liquid smoke flavourings were tested as non-extracted and extracted. To assess the potential drivers of toxicity, we used two different solvents. The battery of in vitro methods covered estrogenicity, androgenicity, oxidative stress, aryl hydrocarbon receptor activity and genotoxicity. The non-extracted samples were tested at concentrations 0.002 to 1 μL liquid smoke flavouring/mL culture medium, while extracted samples were tested from 0.003 to 200 μL/mL. Genotoxicity was observed for nearly all non-extracted and all hexane-extracted samples, in which the former had higher potency. No genotoxicity was observed for ethyl acetate-extracted samples. Oxidative stress was activated by almost all extracted and non-extracted samples, while approximately half of the samples had aryl hydrocarbon receptor and estrogen receptor activities. This study used effect-based methods to evaluate the complex mixtures of liquid smoke flavourings. The increased bioactivities seen upon extractions indicate that non-polar chemicals are driving the genotoxicity, while polar substances are increasing oxidative stress and cytotoxic responses. The differences in responses indicate that non-extracted products contain chemicals that are able to antagonize toxic effects, and upon extraction, the protective substances are lost.

**Supplementary Information:**

The online version contains supplementary material available at 10.1007/s00204-021-03190-1.

## Introduction

While smoking of foods traditionally has been performed mainly as a mean of preservation, it is today also used to create foods with a desired flavour of smoke. This has resulted in the development of smoke flavouring products, which are adding smoke flavour to food without actual smoking of the food item. Smoke flavourings are produced by thermal treatment of wood in the absence of oxygen (pyrolysis), followed by condensation of the vapours and fractionation of the liquid products, resulting in a complex mixture of compounds (EFSA FAF Panel 2021; Sikorski 2004). It is well known that this process also produces hazardous compounds that could pose a risk to public health, e.g., polycyclic aromatic hydrocarbons (PAHs) like benzo[a]pyrene (BaP) (Šimko 2018; Yabiku et al. 1993). Smoke flavourings are specifically regulated according to Regulation (EC) No 2065/2003, which focuses on the usage of smoke flavourings on or in food items (European Parliament, Council of the European Union 2003). There are currently ten primary smoke flavourings authorized to be used in or on food items (Council of the European Union 2013). Primary products are the primary smoke condensates and primary tar fractions, which are further processed to produce the smoke flavourings applied in food. European Food Safety Authority (EFSA) recently issued an updated guidance document for application on smoke flavouring primary products (EFSA FAF Panel 2021). The initial toxicity studies needed focus on potential genotoxic properties of the smoke flavours, and a tiered approach is applied by combining in silico*, *in vitro and in vivo evaluations of genotoxic properties. In addition, tier I safety data for developmental and reproductive toxicity are required for new authorizations (EFSA FAF Panel 2021, Appendix E). In 2010–2012, EFSA published a number of safety assessments of smoke flavouring primary products where they concluded that there were safety concerns for the proposed uses and levels for several products, whereas others were of no safety concern (EFSA Panel on Food Contact Materials 2011a, b, 2012).

The smoke flavouring primary products, evaluated by EFSA, are mainly used in the food industry. However, there are also smoke flavouring products commercially available directly to consumers. The products available on the market and the primary products are supposedly produced in a similar manner by pyrolysis, but from different sorts of woods. To differentiate the products, we have tested in this study from the smoke flavouring primary products evaluated by EFSA, the tested products will hereafter be called: liquid smoke flavourings.

Liquid smoke flavourings are characterized by having a high variability and complex chemical composition with limited information on toxicity of individual chemical constituents, a large number of unidentified chemicals, and potential interaction between chemicals in the mixture (EFSA FAF Panel 2021). Thus, alternative methods for toxicity testing would be useful (Montazeri et al. 2013). Effect-based methods, often based on cultured mammalian cells that have been modified to respond to key molecular events early in toxicity pathways, have proven to be valuable to evaluate highly complex mixtures (Escher et al. 2021; Rosenmai et al. 2017; Selin et al. 2021).

In this study, we used a panel of in vitro bioassays to evaluate effects of hazardous chemicals in ten commercially available liquid smoke flavourings. The liquid smoke flavourings were tested as non-extracted and extracted, and potential drivers of toxicity were tested by using two different solvents for the extraction. Endpoints covered were estrogenicity, androgenicity, oxidative stress, aryl hydrocarbon receptor activity (AhR), and genotoxicity.

## Materials and methods

### Liquid smoke flavourings

Ten liquid smoke flavourings marketed to consumers were purchased from different online stores, and were produced from apple, hickory, mesquite, oak, and pecan wood (Table 1). All samples were used within their expiration date. Information of the ingredients and recommended doses are provided in Table SI-2. No information on the flavouring ingredient other than the wood was given on the product itself.

**Table 1:** Sample ID analysed as non-extracted and extracted liquid smoke flavourings

Smoke flavouring	Extraction method			
	Non-extracted	SPE (Hex)	LLE (Hex)	LLE (EA)
Apple	A1	A1 SPE		
Hickory	H1	H1 SPE		
	H2	H2 SPE		
	H3	H3 SPE		
	H4	H4 SPE	H4 Hex	H4 EA
	H5	H5 SPE	H5 Hex	H5 EA
Mesquite	M1	M1 SPE		
	M2	M2 SPE		
Oak	O1	O1 SPE		
Pecan	P1	P1 SPE		

*Hex* hexane, *EA* ethyl acetate

For the non-extracted liquid smoke flavouring, 2 mL was filtered using a 0.22 μm syringe. Thereafter, the samples were transferred into Eppendorf tubes and stored at + 4 °C until analysis.

The liquid smoke flavourings were also extracted by two different solvents, namely hexane (Hex, log *K*_ow_ = 3.8) and ethyl acetate (EA, log *K*_ow_ = 0.7), to investigate to what extent polar or non-polar substances are driving the toxic effects.

Samples were extracted with either solid-phase extraction (SPE) or liquid–liquid extraction (LLE), the latter using both hexane and ethyl acetate. We wanted to compare the simpler and more traditionally used extraction method LLE against the more automated SPE method. SPE was performed with Oasis HLB 20 cc cartridges that are able to extract a wide range of compounds with pH ranging from 0 to 14. The extraction procedures are described in the Supplementary Information (SI-1, Sect. 1). In short, samples were either extracted by SPE with hexane or LLE using either hexane or ethyl acetate (Table 1). After extraction, samples were evaporated to dryness using nitrogen and resuspended in 0.5 mL of DMSO before being transferred into Eppendorf tubes for bioanalysis. Hickory samples 1, 2, and 5 were not dissolved in DMSO due to their oily composition and were diluted in cell culture media instead of DMSO. Only two samples were successfully extracted through LLE, namely hickory sample 4 and 5, since a clear separable solvent phase was not obtained for the rest of the samples. The concentrations of extracted samples are given as μL liquid smoke flavouring used for the extraction per mL cell culture medium, to enable a comparison of effect concentrations between non-extracted and extracted samples. The extracted samples were stored at – 20 °C.

### Effect-based in vitro methods

Effect-based tests that covered reactive, non-specific, and specific modes of actions were applied (Escher et al. 2021). The methods assessed activation of AhR, androgenicity (AR), estrogenicity (ER), oxidative stress (Nrf2), and genotoxicity (micronucleus test) (Table 2). Detailed information of the methods is presented in the Supplementary Information (Table SI-1).

**Table 2:** Summary of the effect-based in vitro methods

In vitro method	Cell line	Reference compound	Concentration (µM)
Cytotoxicity	All cell lines mentioned below	N/A	N/A
Aryl hydrocarbon receptor activity	DR-EcoScreen	2,3,7,8-Tetrachlorodibenzodioxin (TCDD)	1 × 10^–8^ to 3 × 10^–4^
Androgen receptor agonistic activity	AR-EcoScreen GR-KO M1	Dihydrotestosterone (DHT)	1 × 10^–9^ to 1 × 10^–3^
Androgen receptor antagonistic activity	AR-EcoScreen GR-KO M1	Hydroxyflutamide (OHF)	1 × 10^–5^ to 1 × 10^1^
Estrogen receptor agonistic activity	VM7Luc4E2	Estradiol (E2)	4 × 10^–7^ to 4 × 10^–4^
Oxidative stress response	MCF7 AREc32	Tert-Butylhydroquinone (tBHQ)	8 × 10^–1^ to 2.5 × 10^1^
Micronucleus test	TK6	Mitomycin C* (MMC)	1 × 10^–1^ and 2 × 10^–1^

*MMC was used as a positive control

For all assays, a specific reference compound was used as a standard to validate each run. The vehicle controls consisted of cell medium for the non-extracted smoke flavourings and hickory samples 1, 2, as well as 5, and DMSO was used for the remaining extracted samples.

Cytotoxicity was evaluated by MTS and ATPase assay, as described in the Supplementary Information (SI, Sect. 1.5), and by ethidium monoazide stain (EMA) in the genotoxicity (micronucleus) assay.

The non-extracted smoke flavouring samples were tested at concentrations 0.002–1 μL liquid smoke flavouring/mL cell culture medium and the extracted samples were tested at concentrations from 0.003 to 200 μL liquid smoke flavouring/mL cell culture media. The concentrations used in the bioassays were decided from the effects on cytotoxicity to ensure that bioactivity was assessed at non-cytotoxic concentrations. Samples were analysed in either twofold or fivefold dilutions.

### Data evaluation

Cell viability results were normalized to the vehicle control, which was set as 100%. Samples causing more than 20% reduction were considered cytotoxic, except for the micronucleus test where the cytotoxicity limit was defined as fourfold increase in %EMA-positive events compared to the vehicle control.

For nuclear receptor agonistic response, the activity was first normalized to the vehicle control, and then normalized to the maximum (max) effect of the standard. The antagonistic receptor activities of samples were normalized to vehicle controls without DHT, followed by normalization to the max effect of the vehicle control exposed to DHT. Standard curves for the nuclear receptors were created in GraphPad Prism 9 Software (San Diego, California, USA) using non-linear (log logistic) sigmoidal curve fit. For oxidative stress response, the activity was normalized to the vehicle control, since no max effect can be reached (Escher et al. 2018). The response was therefore fitted to a linear regression dose–response curve.

The limit of detection (LOD) was calculated as three times the standard deviation of the vehicle control. The cut-off limit was based on the LOD and used to define a sample as bioactive. The cut-off was set as the even number above the LOD for agonistic activity and below the LOD for antagonistic activity (Escher et al. 2018, Table SI-1).

The cut-off was set at 70% for antagonistic samples; thus, samples with activities at or below 70% were considered bioactive. The effect concentration 20% (EC_20_) or inhibitory concentration 30% (IC_30_) was calculated for agonistic and antagonistic activity for all bioactive samples, respectively.

Effect concentration induction ratio 1.5 (EC_IR1.5_) was calculated for samples in the oxidative stress Nrf2 assay. Nrf2 activity was presented as fold change compared to the vehicle controls.

For genotoxicity, the micronucleus formation data were analysed in GraphPad Prism 9 using one-way ANOVA with Dunnett’s multiple comparison test. Samples were defined as bioactive if the responses were statistically significant compared to the vehicle control value (*p *value < 0.05).

The bioanalytical equivalent concentration (BEQ) was calculated, to relate the effect of the sample to a known reference compound, according to the following formula (Escher et al. 2015):

$$\mathrm{BEQ}= \frac{{\mathrm{EC}}_{20}\mathrm{ or} {\mathrm{EC}}_{\mathrm{IR}1.5 }\mathrm{or }{\mathrm{IC}}_{30} (\mathrm{reference compound})}{{\mathrm{EC}}_{20}\mathrm{ or }{\mathrm{EC}}_{\mathrm{IR}1.5}\mathrm{ or }{\mathrm{IC}}_{30} (\mathrm{sample})}.$$

The BEQ was then multiplied with the recommended serving size from the manufacturers to retrieve the estimated exposure in bioequivalents of reference compound per serving size/portion. If the recommended serving size was not stated, it was assumed to be 5 mL (Table SI-3). For the manufacture that stated that the recommended serving size was a few drops, we estimated that one drop was 0.05 mL and that three drops would be representative as the serving dosage.

## Results

### Cytotoxicity

Cell viability was investigated after 24 h exposure of MCF7 AREc32, DR-Ecoscreen, VM7Luc4E2, and AR-EcoScreen with glucocorticoid receptor knockout mutant 1 (GR-KO MI) cells to liquid smoke flavourings (Figs. SI-1, SI-3, SI-5, SI-7). Treatments that reduced the viability with more than 20% were considered cytotoxic and were excluded from further testing.

Non-extracted samples exhibited higher cytotoxicity in comparison to all extracted samples. For the majority of the non-extracted samples, cytotoxicity was observed at the highest concentration tested (Figs. 2A, SI-1, SI-3, SI-5, SI-7). Non-extracted hickory samples 2, 4, and 5 retrieved the highest potency of all tested samples in MCF7 AREc32, DR-EcoScreen GR-KO M1, VM7Luc4E2, and AR-EcoScreen cell lines (Figs. SI-1, SI-3, SI-5, SI-7).

A similar trend of cytotoxicity was seen for ethyl acetate LLE samples, where higher potencies were obtained for ethyl acetate-extracted samples than for hexane-extracted samples (Figs. 2A, SI-1C, SI-3C SI-5C, SI-7C).

SPE samples exhibited varying cytotoxicity, although to a considerable lower degree compared to the non-extracted samples (Figs. 2A, SI-1, SI-3, SI-5, SI-7).

### Oxidative stress (Nrf2)

**Fig. 1:**
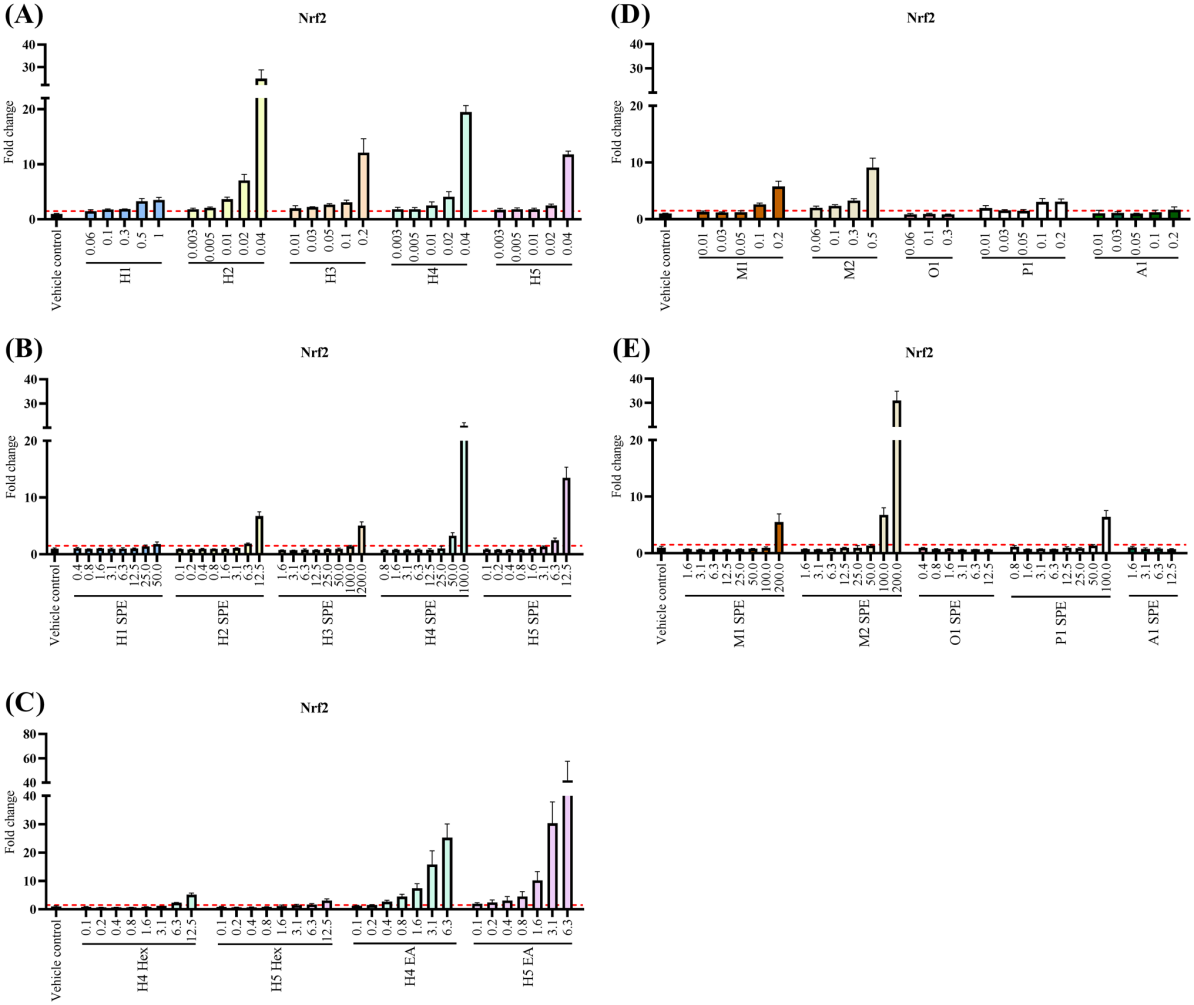
Nrf2 response (fold change compared to control) upon 24 h exposure of MCF7 AREc32 cells to liquid smoke flavourings: non-extracted (**A**, **D**), SPE extracted (**B**, **E**), and LLE extracted (**C**). Concentrations on the *x*-axis are expressed as μL liquid smoke flavouring/mL cell culture medium. Data illustrate mean ± SD (*n* = 4), and the dotted line represents the induction ratio of 1.5-fold change, defined as the cut-off limit of bioactivity

Oxidative stress, measured as Nrf2 activity, was induced by all non-extracted hickory-smoke product samples 1–5 in a dose-related manner (Fig. 1A). Highest potency was obtained for H2, H4 and H5. H2 was the most potent of all non-extracted samples tested, causing a 25-fold induction at a concentration of 0.04 μL/mL. This specific sample was even bioactive at the lowest concentration of 0.003 μL/mL (Fig. 1A).

Nearly all SPE samples (8/10) were bioactive at the highest concentration tested, however, at considerable higher concentrations (12.5–200 μL/mL) than in the non-extracted samples. Mesquite sample 2 caused oxidative stress to the highest degree, reaching a 31-fold induction at 200 μL/mL (Fig. 1E). LLE samples extracted with ethyl acetate induced Nrf2 with a higher potency than SPE extracted (Fig. 1C). Hickory sample 5, extracted with ethyl acetate, showed the highest activity and was bioactive at all concentrations tested, ranging from 0.1 to 6.3 μL/mL (Fig. 1C). In contrast, samples extracted with hexane showed a very low induction of Nrf2 (Fig. 1C). Hickory sample 4 exhibited a similar Nrf2 efficacy when tested non-extracted, extracted through SPE and LLE with ethyl acetate, however, at different concentrations, 0.04, 100, and 6.3 μL/mL, respectively (Fig. 1A–C). A similar oxidative stress response was seen for non-extracted and SPE mesquite sample 1 at the highest concentration tested, although the former being 1000 times less concentrated (Fig. 1D, E).

The linear dose–response of the standard tBHQ is presented in the Supplementary Information (Fig. SI-2). For all non-extracted bioactive samples, defined by the cut-off limit of 1.5-fold change, BEQ values were calculated as mg tBHQ equivalent concentrations (eq) per serving size/dose. The BEQ values ranged from 0.9 to 452 mg tBHQeq per 5 mL sample (Table SI-3). Hickory sample 2, 4, and 5 retrieved the highest BEQ values of 452, 384, and 345 mg tBHQeq/5 mL sample, respectively (Table SI-3).

### Genotoxicity

**Fig. 2:**
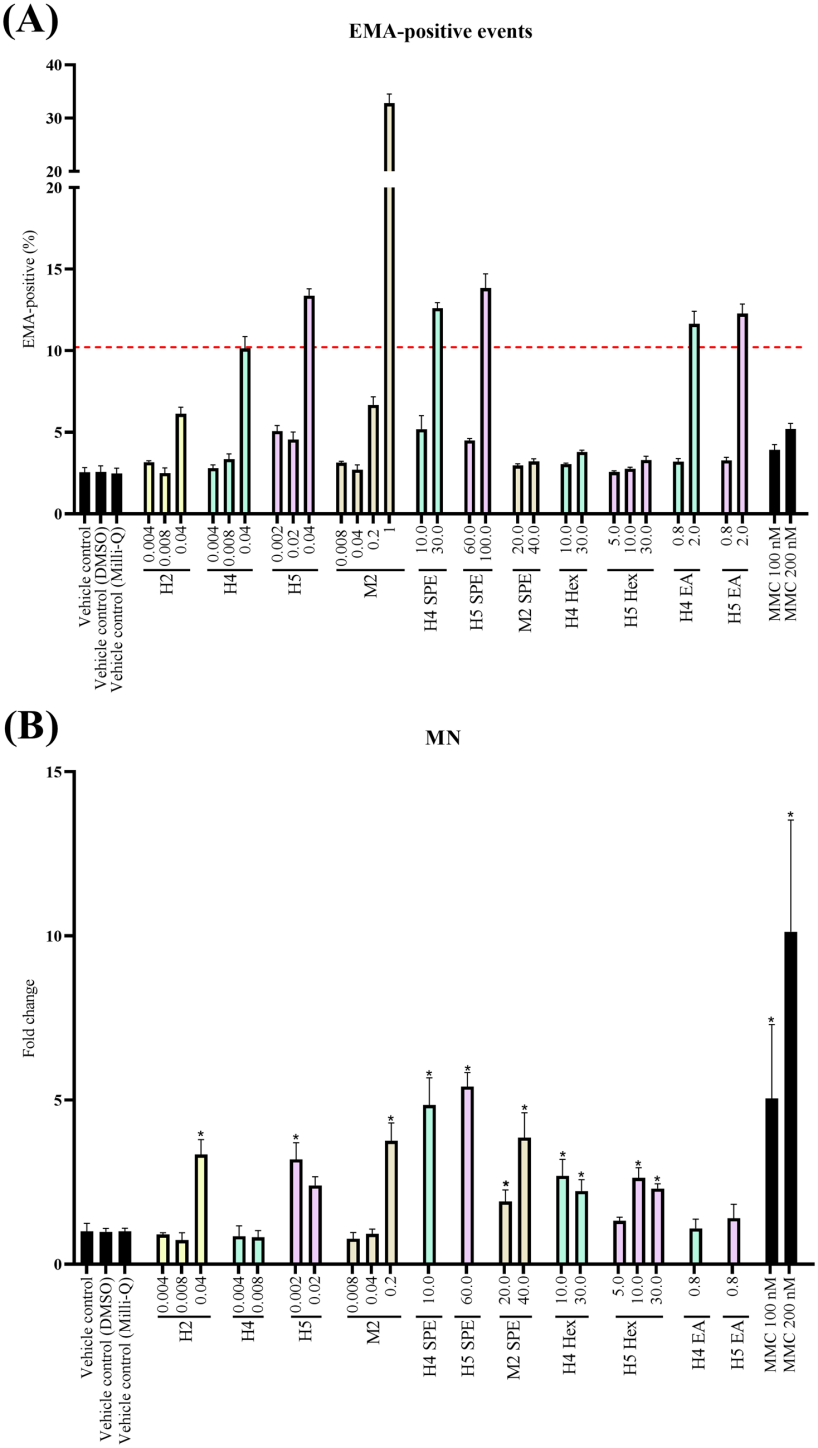
Cytotoxic and genotoxic response (fold change of micronuclei events compared to control) upon 24 h exposure of TK6 cells to liquid smoke flavourings: cytotoxicity (**A**) and micronuclei events (**B**). Concentrations on the *x*-axis are expressed as μL liquid smoke flavouring/mL cell culture medium. The graph demonstrates mean ± SD, *n* = 12 for controls and *n* = 4 for samples. Mitomycin C (MMC) was used as a positive control at concentrations 100 and 200 nM. Samples that were statistically significantly different from the control are indicated with an asterisks (*p value < 0.05)

Samples inducing oxidative stress to a high degree were investigated in the micronucleus test (MN) (Fig. 2). Liquid smoke samples detected as cytotoxic, as indicated by a fourfold increase in % EMA events compared to the control, were excluded for MN assessment (Fig. 2A).

The non-extracted tested hickory sample 2, 5 and mesquite sample 2 showed a statistically significant increase in the micronuclei formation (Fig. 2B). In agreement with the oxidative stress assay, hickory sample 4 and 5 extracted with SPE also caused a statistically significant increase in the genotoxic response (Fig. 2B). Extraction with ethyl acetate did not affect the micronuclei formation. Genotoxicity was observed for both non-extracted and SPE mesquite sample 2, but the potency was higher in the non-extracted sample (Fig. 2B).

The positive control mitomycin C caused an elevated genotoxic response in a dose-dependent manner, in which the highest concentration of 200 nM caused the highest MN formation.

### AhR activity

**Fig. 3:**
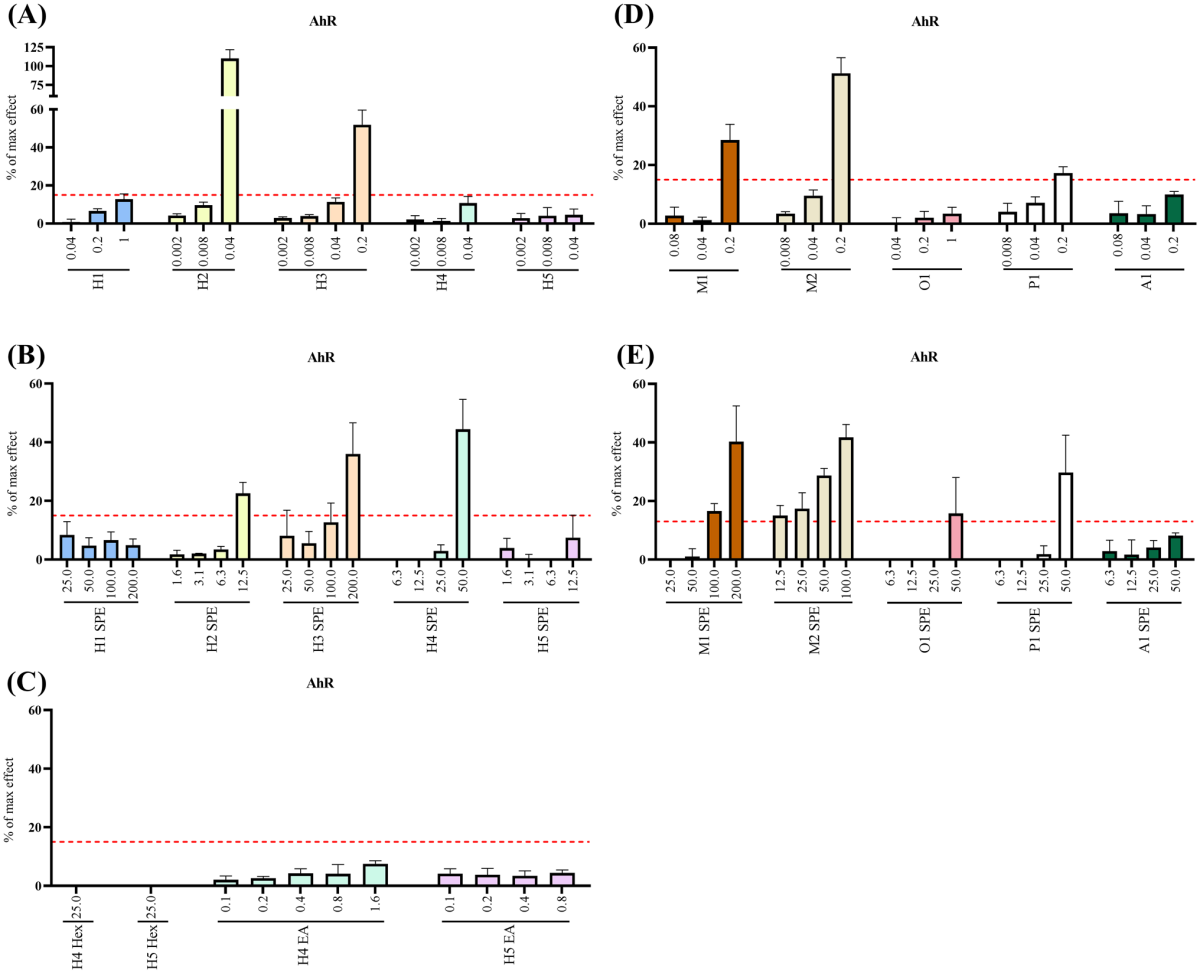
AhR activity (% of max effect) after 24 h exposure of DR-EcoScreen cells to liquid smoke flavourings: non-extracted (**A**, **D**), SPE extracted (**B**, **E**) and LLE extracted (**C**). Concentrations on the x-axis are expressed as μL liquid smoke flavouring/mL cell culture medium. Data illustrate mean ± SD (*n* = 4) and the dotted line represents the % max effect of 15, defined as the cut-off limit of bioactivity

Activation of AhR, defined by the cut-off limit of 15% of max effect, was observed for both non-extracted and SPE extracted samples. The sample being the most potent and having the highest efficacy was hickory sample 2 of all non-extracted samples, while hickory sample 4 was the most potent of all extracted samples (Fig. 3A, B). The highest concentration of SPE extracted hickory sample 2 had 23% of max effect and 40% with hickory sample number 4 (Fig. 3B). Non-extracted and SPE extracted mesquite sample 1 and 2 induced AhR activity even at the lower tested concentrations (Fig. 3D, E). The AhR response drastically increased upon SPE extraction, whereas it remained inactive when tested non-extracted or extracted with hexane and ethyl acetate through liquid–liquid (Fig. 3A–C). Still, worth mentioning is that the effect for the majority of the samples was only visible at the highest non-cytotoxic concentration. The only exceptions were for SPE mesquite sample 1 and 2 (Fig. 3E). Hickory sample 1, 5 and apple sample 1 remained inactive when tested non-extracted and extracted (Fig. 3A–E). Pecan sample 1 evoked a higher AhR response upon SPE extraction, likely due to the higher concentration used and the activity reached a max effect of 30% (Fig. 3E).

TCDD was used as standard and the non-linear dose response is shown in the Supplementary Information (Fig. SI-4). The BEQ values ranged from 14,000 to 300,000 pg TCDDeq per 5 mL sample for the non-extracted samples, in which hickory sample 2 obtained the highest BEQ value (Table SI-3).

### Estrogenicity

We observed estrogenic activity in three out of ten non-extracted samples (Figs. SI-6A, 6D). The activity was only seen at the highest concentration tested for hickory sample 1 and 2, as well as mesquite sample 2.

No ER activity was observed for extracted hickory sample 1 and 2 (Fig. SI-6B). On the contrary, mesquite sample 2 had a drastically increased activity, between 60 and 108% of the max effect, after SPE extraction (Figs. SI-6B, 6E).

ER activity was highly increased in several of the extracted products, this was especially true for SPE extracted samples (Figs. SI-6B, 6E). When comparing the extraction techniques, samples extracted with SPE elicited higher estrogenic response for hickory sample 4 compared to LLE hexane samples (Figs. SI-6B, SI-6C).

SPE caused a higher induction, likely explained by being more concentrated (Figs. SI-6B, SI-6C). Hickory sample 4 extracted with ethyl acetate did not induce estrogenicity, in comparison to the hexane extractions which showed a strong estrogenic response. On the other hand, hickory sample 5 exerted no response as non-extracted or SPE extracted, while LLE extraction by hexane and ethyl acetate caused an elevated response of the estrogen receptor (Figs. SI-6C).

E2 was used as a standard and the non-linear dose–response curve is found in the Supplementary Information (Fig. SI-6F). Only one BEQ value was obtained for the non-extracted samples, which was 1.6 ng E2eq/5 mL for mesquite sample 2, as the remaining samples either remained inactive or were below the detection limit (Table SI-3).

### Androgenicity

The non-extracted and SPE hickory samples did not activate the androgen receptor, defined by the cut-off limit of 4% of max effect (Figs. SI-8A, SI-8B). The lack of response could be explained by the usage of low concentrations, as higher concentrations exerted cytotoxicity. Nevertheless, upon SPE extraction, the oak sample 1 and pecan sample 1 elicited agonistic response of the androgen receptor (Figs. SI-8D, SI-8E). Furthermore, the agonistic response of hickory sample 5 drastically increased in a dose-related manner after LLE with hexane, but it remained inactive when extracted with ethyl acetate (Fig. SI-8C).

No antagonistic mode of action on the androgen receptor was observed when cells were exposed to the non-extracted liquid smoke flavourings (Figs. SI-9A, SI-9D). A few samples exhibited antagonistic effects, but the sudden drop in activity suggests that these effects more likely can be explained by undetected cytotoxicity and should therefore be interpreted with caution (Figs. SI-9B, SI-9E). A similar profile of antagonistic effect was seen for hickory sample 4 that was liquid–liquid extracted with ethyl acetate (Fig. SI-9C).

The non-linear dose–response curves of DHT and OHF are available in the Supplementary Information (Figs. SI-8F, 9F). No BEQ values were obtained for agonistic and antagonistic androgen receptor response (Table SI-3).

## Discussion

In this study, we used a panel of effect-based methods to retrieve information on potential toxicity of the mixture of chemicals that defines liquid smoke flavourings. The specific endpoints studied were oxidative stress, genotoxicity, aryl hydrocarbon, estrogen, and androgen receptor activities in addition to general cytotoxicity.

A high cytotoxicity was observed in all but two of the non-extracted samples. For several of the samples, cytotoxicity was already seen at 1 μL liquid smoke flavouring per 1 mL cell culture medium, and for some even at 0.2 μL/mL. Cytotoxicity was considerably reduced after SPE and LLE with hexane, thus allowing higher concentrations to be tested, and therefore, higher effects were seen in comparison to non-extracted products. The results indicate that cytotoxicity mainly originates from polar substances. This is further supported by the higher cytotoxicity in samples after LLE with ethyl acetate, where cytotoxicity was almost as high as in the non-extracted samples. The results emphasize the impact of extraction procedure in bioanalysis (Abbas et al. 2019).

Exposure of cells to liquid smoke flavourings induced oxidative stress response, determined as Nrf2 activity. All hickory and the two mesquite samples induced oxidative stress especially in non-extracted but also in extracted samples. Extraction procedure had a main impact on the oxidative stress response, and induction of Nrf2 activity was pronounced upon extraction with ethyl acetate, supporting the suggestion that polar substances are main drivers of oxidative stress, as discussed above for cytotoxicity. As oxidative stress may be associated with genotoxicity, four of the samples which induced Nrf2 activity were tested for genotoxic potential by a micronucleus test. Non-extracted samples had a higher potency compared to hexane-extracted samples, except for one non-extracted sample (H4), which was not genotoxic at non-cytotoxic concentrations. Interestingly, ethyl acetate-extracted samples did not increase micronuclei formation, which may be explained by the low concentrations used as higher concentration caused toxicity, or by the fact that polar substances do not drive genotoxicity.

Previous studies have shown increased DNA single-strand breaks (Ohshima et al. 1989), altered pyloric glands in rats after oral exposure to hickory-smoke condensate (Shichino et al. 1992) and mutation in human lymphocytes in vitro after exposure to aqueous wood smoke flavourings (Braun et al. 1987). A more recent study confirmed the genotoxic potential of commercially available liquid smoke flavourings in a human p53 reporter gene cell line, and reported higher p53 response in hickory than mesquite samples (Hossain et al. 2013). Additionally, increased γ-H2AX, p21 and p53 protein levels were detected. However, other studies failed to detect genotoxicity or obtained inconclusive results in the Ames test (Braun et al. 1987; Putnam et al. 1999). The lack of effect in the Ames test may be explained by the low sensitivity of the test and/or usage of different smoke flavoured products (Kirkland et al. 2014). PAHs are generated during the smoke formation and are thought to covalently bind to protein and nucleic acids, forming DNA adducts that may be carcinogenic (Luo et al. 2008; Oz 2020; Šimko 2011).

The formation of PAHs is of human health concern and has to be analysed for authorization of liquid smoke flavourings (Commission Regulation EC No 627/2006 2006). Metabolic activation is needed for PAHs to exert DNA damaging effects. In this study, we did not include a metabolic activation system and the results therefore suggest that the genotoxicity observed is mediated through other chemicals. Furthermore, the specificity of TK6 cells to distinguish between clastogen and aneugen modes of action seems to be lower in comparison to when other cell lines are used (Bryce et al. 2011; Smart et al. 2020). The results in this study together with previous studies show that a variety of commercially available liquid smoke flavourings may have genotoxic properties in vitro, which needs to be further investigated.

AhR activity was induced in five of the ten non-extracted samples and in seven of the SPE samples, although at much higher concentrations. This was most obvious for hickory sample 2, where the non-extracted sample had the highest efficacy of all tested liquid smoke samples, and the activity was drastically reduced after SPE extraction. AhR activity can be induced by numerous chemicals, for example by PAHs (Boonen et al. 2020).

Apart from a single positive sample and two at the cut-off level, no ER activity was observed in the non-extracted samples. However, higher concentrations could be tested than of the non-extracted samples due to cytotoxicity, and seven of the SPE samples exhibited estrogenic activities. Boonen et al. (2020) reported ER activity by BaP in bioassays. No AR activities, agonistic or antagonistic, were detected in the non-extracted samples, while two of the SPE samples were active in the highest concentration. PAHs have been shown to induce antagonistic androgen receptor activity in water samples (Xu et al. 2019).

Bioactivities varied widely between the various products. Some products exhibited no or a low activity in all assays (H1, O1), while others had a high activity in several of the assays (H2, M2). The bioactivities depend on the concentrations of the individual bioactive compounds and interactions between the compounds in the mixture, which are unknown factors. Wood type, burning conditions, purification, pH-, total acid, chemical, and water content are factors influencing the chemical composition of smoke flavourings (Budaraga et al. 2016; Sikorski 2004; Šimko 2005). The commercially available liquid smoke flavourings investigated in the present study had limited information on manufacturing and identity, compared to the registered smoke flavourings (Council of the European Union 2013). However, it is supposed that the smoke flavourings in general should be regarded as safer than smoke products generated directly from the traditional smoking procedure, as toxic chemicals can be removed during the filtration and purification processes (European Parliament, Council of the European Union 2003).

The bioequivalent concentrations corresponding to the observed bioactivity for the non-extracted products in each assay was calculated and expressed as bioequivalents of the reference compound per serving size, to allow a comparison to the estimated intake via food or drinking water. For estrogenic activity, the only sample with a BEQ value was M2, corresponding to 1.6 ng E2eq per serving. This can be compared to the WHO benchmark value for drinking water of 1 ng E2/L (WHO 2017). The daily consumption of drinking water is estimated to 2 L, which means that the exposure of E2eq from one serving size of M2 is below the benchmark value of E2 in drinking water.

For oxidative stress, the BEQ values ranged from 0.9 to 452.0 mg tBHQeq/serving. This value can be compared to the acceptable daily intake (ADI) of tBHQ provided by EFSA, which is 0.7 mg/kg bw/day, corresponding to 49 mg/day at a body weight of 70 kg (EFSA 2004). Six of the ten liquid smoke flavourings resulted in intakes above the ADI for one serving size, of which hickory samples 2, 4, and 5 had the highest BEQ values.

The calculated guidance value for AhR activity was in this case not appropriate, as the liquid smoke products obviously is not induced by dioxins or planar PCBs, but rather by other chemicals with different toxicokinetics and toxicodynamics. The calculated TCDD equivalents from the liquid smoke flavourings ranged from 14,000 to 300,000 pg per serving, and greatly exceeded the tolerable weekly intake (TWI) established by EFSA of 2 pg/kg body weight, corresponding to 140 pg/person/week (EFSA Panel on Contaminants in the Food Chain (CONTAM) et al. 2018).

Information on potential toxic effects of the commercially available liquid smoke flavourings is scarce. In contrast, toxicity of smoke products in E-cigarettes has attracted more attention. Smoke flavourings as food additives and in E-cigarettes are both based on the same concept, namely to remove the most toxic substances produced from natural combustion, while retaining flavouring compounds. Cell-based bioassays have been used for hazard evaluation of cigarette smoke constituents, but we are not aware of a similar approach for hazard evaluation of liquid smoke flavourings (Barhdadi et al. 2021; Moore et al. 2020; Rudd et al. 2020; Stabbert et al. 2017). Even if the route of exposure differs between E-cigarettes and liquid smoke flavourings, both will reach the systemic circulation after absorption. Rudd et al. (2020) reported that E-cigarettes should be considered as a safer option to cigarette smoke, which likely can be explained by the fact that the flavour and nicotine are received through aerosolization of E-cigarettes, compared to burning of tobacco in cigarette smoke, allowing fewer toxicants to be formed. It was concluded that less cytotoxicity in the neutral red uptake (NRU) assay and no mutagenicity (Ames test) or genotoxicity (MN test) was seen for E-cigarettes, compared to the reference cigarette. However, it is not agreed within the research field that e-cigarettes should be considered as safer, as these liquids may contain genotoxicants (Barhdadi et al. 2021).

The same controversy can be said for liquid smoke flavourings in comparison to the traditional smoking of food. A similar approach to use and generate in vitro data of E-cigarettes would be recommended to be applied to liquid smoke flavourings (Moore et al. 2020).

In this study, we have tested ten commonly used liquid smoke flavourings and used two different solvents to investigate if polar or non-polar substances are driving the toxic effects. The increased bioactivities upon extraction indicate that non-polar substances are driving the genotoxicity, whereas polar substances are driving the oxidative stress and cytotoxicity. The usage of effect-based methods allowed testing of the complex whole mixture, enabling us to study interactive effects of the product. Findings in this study indicate that liquid smoke flavourings contain compounds with hazardous properties and to ensure that these widely used products are safe further studies should be carried out.

## Supplementary Information

Below is the link to the electronic supplementary material.

Supplementary file1 (DOCX 2677 KB)

## Data Availability

All data are included in the manuscript or in the Supplementary Material. Further data or information will be supplied upon request.

## References

[CR1] Abbas A, Schneider I, Bollmann A (2019). What you extract is what you see: optimising the preparation of water and wastewater samples for in vitro bioassays. Water Res.

[CR2] Barhdadi S, Rogiers V, Deconinck E, Vanhaecke T (2021). Toxicity assessment of flavour chemicals used in e-cigarettes: current state and future challenges. Arch Toxicol.

[CR3] Boonen I, Van Heyst A, Van Langenhove K (2020). Assessing the receptor-mediated activity of PAHs using AhR-, ERα- and PPARγ- CALUX bioassays. Food Chem Toxicol.

[CR4] Braun AG, Busby WF, Jackman J, Halpin PA, Thilly WG (1987). Commercial hickory-smoke flavouring is a human lymphoblast mutagen but does not induce lung adenomas in newborn mice. Food Chem Toxicol.

[CR5] Bryce SM, Avlasevich SL, Bemis JC, Dertinger SD (2011). Miniaturized flow cytometry-based CHO K1 micronucleus assay discriminates aneugenic and clastogenic modes of action. Environ Mol Mutagen.

[CR6] Budaraga IK, Arnim A, Marlida Y, Bulanin U (2016). Liquid smoke production quality from raw materials variation and different pyrolysis temperature. Int J Adv Sci Eng Inf Technol.

[CR7] Council of the European Union (2013) Commission Implementing Regulation (EU) No 1321/201

[CR8] Commission Regulation EC No 627/2006 (2006) Implementing Regulation (EC) No 2065/2003 of the European Parliament and of the Council as regards quality criteria for validated analytical methods for sampling, identification and characterisation of primary smoke products

[CR9] EFSA Panel CEF (2011). Scientific opinion on the safety of smoke flavour primary product Fumokomp—2011a update. EFSA J.

[CR10] EFSA Panel CEF (2011). Scientific opinion on the safety of smoke flavour primary product Zesti smoke code 10–2011b update. EFSA J.

[CR11] Knutsen HK, Alexander J, Barregård L, EFSA Panel on Contaminants in the Food Chain (CONTAM) (2018). Risk for animal and human health related to the presence of dioxins and dioxin-like PCBs in feed and food. EFSA J.

[CR12] Younes M, Aquilina G, Castle L, EFSA Panel on Food Additives and Flavourings (FAF) (2021). Scientific Guidance for the preparation of applications on smoke flavouring primary products. EFSA J.

[CR13] EFSA Panel on Food Contact Materials, E, Flavourings and Processing Aids (CEF) (2012). Scientific opinion on the safety of smoke flavouring primary product SmokEz C-10 - 2012 Update. EFSA J.

[CR14] Escher BI, Neale PA, Leusch FDL (2015). Effect-based trigger values for in vitro bioassays: reading across from existing water quality guideline values. Water Res.

[CR15] Escher BI, Neale PA, Villeneuve D (2018). The advantages of linear concentration-response curves for in vitro bioassays with environmental samples: linear CRC. Environ Toxicol Chem.

[CR16] Escher BI, Neale PA, Leusch F (2021). Bioanalytical tools in water quality assessment. IWA Publ.

[CR100] European Food Safety Authority (EFSA) (2004). Opinion of the Scientific Panel on food additives, flavourings, processing aids and materials in contact with food (AFC) on a request from the Commission related to tertiary- Butylhydroquinone (TBHQ). EFSA J.

[CR17] European Parliament, Council of the European Union (2003) Regulation (EC) No 2065/2003 of the European Parliament and of the Council of 10 November 2003 on smoke flavourings used or intended for use in or on foods

[CR18] Hossain MZ, Gilbert SF, Patel K (2013). Biological clues to potent DNA-damaging activities in food and flavoring. Food Chem Toxicol.

[CR19] Kirkland D, Zeiger E, Madia F (2014). Can in vitro mammalian cell genotoxicity test results be used to complement positive results in the Ames test and help predict carcinogenic or in vivo genotoxic activity? I. Reports of individual databases presented at an EURL ECVAM Workshop. Mutat Res Toxicol Environ Mutagen.

[CR20] Luo XJ, Chen SJ, Mai BX (2008). Distribution, source apportionment, and transport of PAHs in sediments from the Pearl River Delta and the Northern South China Sea. Arch Environ Contam Toxicol.

[CR21] Montazeri N, Oliveira ACM, Himelbloom BH (2013). Chemical characterization of commercial liquid smoke products. Food Sci Nutr.

[CR22] Moore MM, Clements J, Desai P (2020). Workshop series to identify, discuss, and develop recommendations for the optimal generation and use of in vitro assay data for tobacco product evaluation: phase 1 genotoxicity assays. Appl Vitro Toxicol.

[CR23] Ohshima H, Furihata C, Matsushima T, Bartsch H (1989). Evidence of potential tumour-initiating and tumour-promoting activities of hickory smoke condensate when given alone or with nitrite to rats. Food Chem Toxicol.

[CR24] Oz E (2020). Effects of smoke flavoring using different wood chips and barbecuing on the formation of polycyclic aromatic hydrocarbons and heterocyclic aromatic amines in salmon fillets. PLoS ONE.

[CR25] Putnam KP, Bombick DW, Avalos JT, Doolittl DJ (1999). Comparison of the cytotoxic and mutagenic potential of liquid smoke food flavourings, cigarette smoke condensate and wood smoke condensate. Food Chem Toxicol.

[CR26] Rosenmai AK, Bengtström L, Taxvig C (2017). An effect-directed strategy for characterizing emerging chemicals in food contact materials made from paper and board. Food Chem Toxicol.

[CR27] Rudd K, Stevenson M, Wieczorek R (2020). Chemical composition and in vitro toxicity profile of a pod-based e-cigarette aerosol compared to cigarette smoke. Appl Vitro Toxicol.

[CR28] Selin E, Svensson K, Gravenfors E (2021). Food contact materials: an effect-based evaluation of the presence of hazardous chemicals in paper and cardboard packaging. Food Addit Contam Part A.

[CR29] Shichino Y, Tatematsu M, Ohshima H (1992). Effects of hickory-smoke condensate on development of pepsinogen 1-altered pyloric glands in rats. Food Chem Toxicol.

[CR30] Sikorski ZE, Jensen WK (2004). SMOKING | traditional. Encyclopedia of meat sciences.

[CR31] Šimko P (2005). Factors affecting elimination of polycyclic aromatic hydrocarbons from smoked meat foods and liquid smoke flavorings. Mol Nutr Food Res.

[CR32] Šimko P, Kerry JP, Kerry JF (2011). 19 - Heat and processing generated contaminants in processed meats. Processed meats.

[CR33] Šimko P, Toldrá F (2018). Chapter seven—modern procedures for removal of hazardous compounds from foods. Advances in food and nutrition research.

[CR34] Smart DJ, Helbling FR, Verardo M, Huber A, McHugh D, Vanscheeuwijck P (2020). Development of an integrated assay in human TK6 cells to permit comprehensive genotoxicity analysis in vitro. MRGTEM.

[CR35] Stabbert R, Dempsey R, Diekmann J (2017). Studies on the contributions of smoke constituents, individually and in mixtures, in a range of in vitro bioactivity assays. Toxicol Vitro.

[CR36] WHO (2017) Drinking water Parameter Cooperation Project. Support to the revision of Annex I Council Directive 98/83/EC on the Quality of Water Intended for Human Consumption (Drinking Water Directive). https://ec.europa.eu/environment/water/water-drink/pdf/WHO_parameter_report.pdf. Accessed 11 Aug 2021

[CR37] Xu S, Zhou S, Xing L (2019). Fate of organic micropollutants and their biological effects in a drinking water source treated by a field-scale constructed wetland. Sci Total Environ.

[CR38] Yabiku HY, Martins MS, Takahashi MY (1993). Levels of benzo [a] pyrene and other polycyclic aromatic hydrocarbons in liquid smoke flavour and some smoked foods. Food Addit Contam.

