# Supplementary material for: An in vitro-based hazard assessment of liquid smoke food flavourings
Source: Arch Toxicol. 2021 Nov 20;96(2):601–11. doi: 10.1007/s00204-021-03190-1 (PMC8837572; doi:10.1007/s00204-021-03190-1)
Supplement: Supplementary file 1 — Supplementary file1 (DOCX 2677 KB) [file 204_2021_3190_MOESM1_ESM.docx]

# Supplementary information

**An *in vitro* based hazard assessment of liquid smoke food flavourings**

Erica Selin^1*^, Geeta Mandava^1^, Alexandra-Livia Vilcu^1^, Agneta Oskarsson^1^ and Johan Lundqvist^1^

^1^Department of Biomedical Science and Veterinary Public Health, Swedish University of Agricultural Sciences, Box 7028, SE-750 07 Uppsala, Sweden

*corresponding author: Department of Biomedical Science and Veterinary Public Health, Swedish University of Agricultural Sciences, Box 7028, SE-750 07 Uppsala, Sweden

E-mail address: [erica.selin@slu.se](mailto:erica.selin@slu.se) (E. Selin)

ES: <https://orcid.org/0000-0001-7869-4549>

GM: <https://orcid.org/0000-0003-2834-2259>

AO: <https://orcid.org/0000-0002-3134-7811>

JL: <https://orcid.org/0000-0001-5693-9007>

Table of contents

[1. Section SI-1: Bioanalytical methods information 3](#_Toc67659026)

[1.1 Chemicals and solvents 3](#_Toc67659027)

[1.2 Sample preparation 3](#_Toc67659028)

[1.3 Cell cultures 4](#_Toc67659029)

[1.4 Cell culture exposure 5](#_Toc67659030)

[1.5 Cell viability measurements 5](#_Toc67659031)

[1.6 Effect-based *in vitro* methods 6](#_Toc67659032)

[1.7 Micronuclei formation 7](#_Toc67659033)

Table SI-1 8

Table SI- 2 9

Table SI-3 10

Fig. SI-1 11

Fig. SI- 2 12

Fig. SI- 3 13

Fig. SI- 4 14

Fig. SI- 5 15

Fig. SI- 6 16

Fig. SI- 7 17

Fig. SI-8 18

Fig. SI-9 19

### **Section SI-1: Bioanalytical methods information**

## Chemicals and solvents

Methoxychlor (MeCl, CAS 72-43-5, 98.7%), 5α-androstan-17β-ol-3-one (DHT, CAS 521‑18‑6, ≥97.5%), β-estradiol (E2, CAS 50-28-2, ≥98%), 2,3,7,8-Tetrachlorodibenzo-p-dioxin solution (TCDD, CAS 1746-01-6), tert-Butylhydroquinone (tBHQ, CAS 1948-33-0, 97%), Ethyl acetate (CAS 141-78-6, > ≥99.5%) and dimethyl sulfoxide (DMSO, CAS 67-68-5 >99.9%) were obtained from Sigma-Aldrich. Methanol (CAS 67-56-1, 100%) and hexane (CAS: 110-54-3, ≥97%) were purchased from VWR and Avantor Performance Materials (Poland S.A., Gliwice, Poland), respectively. Milli-Q water® was retained from a Millipore water purification system with a 0.22 µm membrane filter.

## Sample preparation

1. **SPE**

The liquid smoke flavourings were extracted with the solid phase extraction (SPE) cartridges Oasis HLB 20 cc, 1 g (Waters, USA). The cartridges were first pre-conditioned with 30 mL methanol and 10 mL hexane, then 10 mL of the smoke products were added at a speed of 1 drop/sec by the vacuum manifold. Each sample was eluted twice with 10 mL hexane, followed by pooling and evaporated to dryness with nitrogen. Thereafter, the samples were resuspended with DMSO to a final volume of 0.5 mL. Hickory samples 1, 2 and 5 were not dissolved in DMSO as their oily composition prevented evaporation to dryness, resulting in a final volume of 2 mL, 0.5 mL and 0.6 mL, respectively. Fresh dilutions were made in culture media prior to each experiment.

The majority of the samples were enriched by a factor of 20X. Hickory sample 1 retrieved an enrichment factor of 5X and hickory sample 5 had an enrichment factor of 17X. Hexane was used as an SPE solvent control and was extracted in the same way as the liquid smoke flavourings. The samples were stored at -20˚C until analysis.

1. **Liquid-liquid extraction**

Liquid-liquid extraction was performed using two different organic solvents, namely ethyl acetate and hexane. In a Falcon tube, 10 mL of the liquid smoke product and 10 mL of one of the organic solvents were added. The samples were mixed by inversion and left for separation for 24 h at room temperature. After two distinct phases were formed, the organic phase was carefully transferred to a new Falcon tube and evaporated to dryness using nitrogen.

Subsequently, the samples were dissolved in 0.5 mL of DMSO and transferred to an Eppendorf tube for bioanalysis. All samples had an enrichment factor of 20X and were stored at -20˚C until analysis. The samples that were successfully extracted with ethyl acetate and hexane were hickory sample 4 and 5 (4 EA, 5 EA, 4 Hex, 5 Hex), while the remaining samples were separated into three distinct layers making the separation and extraction process very difficult.

## Cell cultures

The mouse hepatoma cell line DR-EcoScreen was obtained from the Japanese Collection of Research Bioresources Cell Bank (JCRB, Osaka, Japan), and used to evaluate aryl hydrocarbon receptor (AhR) agonistic response.

Cells were cultured in Minimum Essential Media α (MEM α), 5% FBS, 1% penicillin‑streptomycin (100 units/mL penicillin, 100 µg/mL streptomycin) and 150 µg/mL Hygromycin B Gold (InvivoGen, USA). Similarly, the experimental medium contained the supplements mentioned above, with the exclusion of Hygromycin B Gold.

The stably transfected Chinese hamster ovary (CHO) cell line AR-EcoScreen with glucocorticoid receptor knockout mutant 1 (GR-KO M1) was used to measure androgen receptor (AR) response (JCRB, Osaka, Japan). Cell culture medium consisted of Dulbecco's Modified Eagle Medium: Nutrient Mixture F-12 (DMEM-F12 medium, Sigma-Aldrich, Irvine, United Kingdom) supplemented with 10% fetal bovine serum (FBS), 1% penicillin‑streptomycin, 1% L-glutamine, 50 µg/mL Zeocin (Invitrogen, CA, USA) and 25 µg/mL Hygromycin B Gold (InvivoGen, CA, USA). The experimental medium consisted of DMEM‑F12 (Sigma-Aldrich), 10% dextran charcoal treated FBS, 1% penicillin-streptomycin and 1% L-glutamine.

VM7Luc4E2 cells, the human breast cancer cell line, was to assess estrogen receptor (ER) activity and was kindly received from M. Denison (University of California, CA, USA). The cell line was maintained in cell culture media containing Roswell Park Memorial Institute 1640 (RPMI 1640) with L‑glutamine, 8% FBS, 0.9% penicillin-streptomycin and the positive selector Gentamicin at concentration 0.55 mg/mL (Gentamicin Sulfate, Lonza). During experiments the medium contained DMEM with 4.5 g/L glucose, 4.5% dextran-charcoal treated FBS, 1.9% L‑glutamine, 0.9% penicillin-streptomycin and 0.38 mg/mL Gentamicin.

Oxidative stress was evaluated by using the stably transfected cell line MCF7 ARE c32. Cells were kindly obtained from R. Wolf (University of Dundee, Nethergate, Scotland), and maintained in DMEM GlutaMAX with 4.5 g/L D-glucose completed, 10% FBS, 1% of penicillin-streptomycin as well as 0.8 mg/mL of the selective antibiotic Geneticin (G418, liquid, Life Technologies). The experimental medium was DMEM GlutaMAX with 4.5 g/L D-glucose, 10% FBS and 1% of penicillin‑streptomycin. Additionally, the micronucleus test was conducted with the human lymphoblastic TK6 cell line (American Type Culture Collection, ATCC, Manassas, VA, USA). The medium used during culture and exposure was RPMI 1640 medium supplemented with L‑glutamine, 10% FBS and 1% penicillin‑streptomycin.

The supplements and mediums were obtained from Gibco, Thermo Fisher Scientific, unless stated otherwise. The cell lines were maintained at 37˚C in 5% CO_2_ in an incubator and medium was changed every second to third day. All adherent cells used 0.05% Trypsin‑EDTA (Gibco, Thermo Fisher Scientific) during passaging of cells. The cell lines were used before passage 30, except for the TK6 cell line.

## Cell culture exposure

The liquid smoke flavourings were assessed for cell viability and activity assays in quadruplicates. The non-extracted smoke products were diluted in cell culture medium and prepared fresh for each experiment, while the extracted products were diluted in DMSO. Cell culture media was used to dilute the SPE extracted samples: hickory sample 1, 2 and 5 due to their oily composition causing incomplete dryness. The vehicle controls were 1% DMSO and cell culture media.

For the non-extracted samples, concentrations used ranged from 0.002 to 1 μL liquid smoke/mL cell culture medium. The highest concentration tested in the activity assays for the extracted samples were considered to be non-cytotoxic and ranged from 0.003 to 200 μL liquid smoke/mL cell culture media.

Each experiment for the viability and activities were done at least two to three times at non‑cytotoxic concentrations.

## Cell viability measurements

Cell viability was measured prior to initiating the activity assays and it was assessed with MTS (Cell Titer 96® AQueous One Solution Cell Proliferation Assay, Promega) for the majority of the assays.

TK6 and VM7Luc4E2 cell lines instead used ethidium monoazide (EMA) stain and ATPase (CellTiter-Glo® Luminescent Cell Viability Assay, Promega), respectively.

For the reporter gene cell lines DR-EcoScreen and AR-EcoScreen GR-KO M1, cells were seeded at a density of 1×10^5^ cells/mL in transparent 384-well plates (Costar® Corning Incorporated). MCF7 AREc32 cells were plated at the density 1.3×10^5^ cells/mL. Following 24 h, cells were exposed to liquid smoke flavourings for 24 h. Thereafter, 10 µL/well of CellTiter 96® AQueous One Solution was added to each well and the plate was left in the incubator for 30 min. Absorbance was measured at 490 nm emission on Spark Multimode Microplate Reader (TECAN, Austria, GmbH) using SparkControl version 3.1 software.

The estrogenic cell line VM7Luc4E2, cells were first cultured in white clear bottom 384‑well plate (Corning) at a density of 4×10^5^ cells/mL and left to incubate for 24 h. The second day consisted of exposure to liquid smoke at the concentrations mentioned above. The third day consisted of adding 25 µL/well of CellTiter‑Glo® Luminescent Cell Viability Assay (Promega) and cells were left for 15 min on a plate shaker. Prior to measurement, cells were left for 2 min and a white adhesive sealing film was adhered at the bottom of the plate. The ATP content of the cells was quantified on Spark Multimode Microplate Reader.

For hexane and ethyl acetate extracted samples, the colour from the extracts was interfering with absorbance and luminescence readings. To deal with this problem, medium was removed after 24 h exposure and cells were washed with PBS before addition of MTS or ATPase.

Lastly, TK6 cells were plated in a transparent round bottom 96-well plate (Sarstedt) with the density of 4×10^5^ cells/mL. Directly after plating, cells were exposed to reference compounds and liquid smoke samples for 24 h. Cells were stained with ethidium monoazide (EMA) for 30 min to distinguish dying and dead cells, in accordance with the instructions obtained from the *In vitro* MicroFlow 96 Well Plate Format Kit (Litron Laboratories, NY, USA). Fluorescence was acquired on FACSVerse 8 colour Flow Cytometer (BD BioSciences, Franklin Lakes, NJ, USA) using BD FacSuite version 1.0.6. software. Data was analysed on FCS Express 5 Flow Research Edition. The limit for cytotoxicity was defined as 4-fold EMA‑positive events compared to the vehicle control. Cells over the cut-off limit were considered as cytotoxic.

## Effect-based *in vitro* methods

Aryl hydrocarbon receptor, androgen, estrogen receptors activation and oxidative stress response were measured using luminescence.

Cells were cultured in a white clear bottom 384‑well plate (Corning) with a total cell count of 1×10^5^ cells/mL for DR‑EcoScreen and AR‑EcoScreen GR-KO M1 cells, 4×10^5^ cells/mL for VM7Luc4E2 and 1.3×10^5^ cells/mL for MCF7 AREc32. After 24 h incubation, cells were exposed to liquid smoke flavourings and reference compounds for 24 h. Subsequently, 10 µL/well of passive lysis buffer (PBL) (Promega) were added causing cells to be lysed. The cells were shaken for 10 – 20 min, before 10 µL/well of Luciferase Assay System (Promega) was added with the automatic injection syringe. The activity of each endpoint was quantitatively measured with luminescence on Spark Multimode Microplate Reader. The reading consisted of a five sec period, where the luciferase solution was injected every other sec. A white adhesive film was attached to the bottom of the plate prior to the reading.

## Micronuclei formation

The TK6 cells were plated in a transparent round bottom 96-well plate at a density of 4×10^5^ cells/mL, and cells were directly exposed to the positive control mitomycin C (MMC) and liquid smoke flavourings for 24 h. Afterwards, cells were stained in accordance with MicroFlow *In vitro* Kit (Litron Laboratories, US). Shortly, EMA stained the cells for 30 min under light to be able to distinguish between dead and dying cells. Cells were thereafter lysed and stained with SYTOX Green to distinguish dying and dead cells from healthy cells. The micronucleus formation was measured using the flow cytometer. A minimum of 5000 events and a maximum of 20 000 gates events were collected for each sample.

**Table SI-1:** Summary of the effect-based parameters for the liquid smoke flavourings.

| **Effect-based method** | **Reference compound** | **EC_IR1.5_*/EC_20_/IC_30_** (μM)** | **Cut-off value** |
| --- | --- | --- | --- |
| Oxidative stress response | tBHQ | 0.2 x 10^1^ | 1.5 fold change |
| AhR agonistic response | TCDD | 7.9 x 10^-7^ | 15% of max effect |
| ER agonistic response | E2 | 9.1 x 10^-7^ | 5% of max effect |
| AR agonistic response | DHT | 2.1 x 10^-5^ | 4% of max effect |
| AR antagonistic response | OHF | 5.0 x 10^-5^ | 70% of max effect |

* EC_IR1.5_ was used for oxidative stress response only
** IC_30_ was used for AR antagonistic response

**Table SI- 2:** Summary of the ingredients and producer for the specific liquid smoke flavourings.

| **Liquid smoke product** | **Sample ID:s** | **Producer** | **Ingredients** | **Recommended dose*** |
| --- | --- | --- | --- | --- |
| Apple | A1/A1 SPE | 3 | Water, apple smoke flavour, vinegar, molasses, caramel colour and salt | 1 tsp (4.5 g) |
| Hickory | H1/H1 SPE | 1 | Liquid smoke, sunflower oil | 0.5-2% of total content (few drops). 1 drop = 0.03 g |
|  | H2/H2 SPE | 2 | Water, hickory smoke concentrate, polysorbate 80 | Not stated. |
|  | H3/H3 SPE | 3 | Water, hickory smoke flavour, vinegar, molasses, caramel colour and salt | 1 tsp (4.5 g)d |
|  | H4/H4 SPE/H4 Hex/H4 EA | 4 | Water, natural hickory smoke concentrate | 1 tsp (5 mL) |
|  | H5/H5 SPE/H5 Hex/H5 EA | 5 | Hickory smoke condensate, water | Not stated. |
| Mesquite | M1/M1 SPE | 3 | Water, mesquite smoke flavour, vinegar, molasses and caramel colour | 1 tsp (4.5 g) |
|  | M2/M2 SPE | 6 | Water, tamari soy sauce (water, soybeans, salt, sugar), natural mesquite smoke flavour, distilled vinegar, sugar, salt, caramel colour, garlic and onion | 1 tsp (5 mL) |
| Oak | O1/O1 SPE | 7 | Organic agave nectar, sugar, water, natural flavourings, acidity corrector: citric acid, caramelized sugar | Not stated. |
| Pecan | P1/P1 SPE | 3 | Water, pecan smoke flavour, vinegar, molasses and caramel colour | 1 tsp (4.5 g) |

* Per serving size.

**Table SI-3:** Summary of the estimated exposure in equivalents (eq) of the respective reference compound per serving size/dose.

| **Sample** | | | **Assays** | | | | |
| --- | --- | --- | --- | --- | --- | --- | --- |
|  | | | Oxidative stress tBHQeq  (mg/5 mL sample) | AhR agonism TCDDeq (pg/5 mL sample) | ER agonism E2eq (ng/5 mL sample) | AR agonism  DHTeq  (mg/5 mL sample) | AR antagonism OHFeq  (mg/5 mL sample) |
| **Non-extracted** | Apple | 1 | 5.3 | IA | IA | IA | IA |
|  | Hickory | 1 | 0.9* | IA | B | IA | IA |
|  |  | 2 | 452.0 | 300 000 | B | IA | IA |
|  |  | 3 | 96.5 | 54 000 | IA | IA | IA |
|  |  | 4 | 380.2 | IA | IA | IA | IA |
|  |  | 5 | 345.1 | IA | IA | IA | IA |
|  | Mesquite | 1 | 51.2 | 23 000 | IA | IA | IA |
|  |  | 2 | 35.7 | 52 000 | 1.6 | IA | IA |
|  | Oak | 1 | IA | IA | IA | IA | IA |
|  | Pecan | 1 | 159.5 | 14 000 | IA | IA | IA |
| Total activity | | | 9/10 | 5/10 | 3/10 | 0/10 | 0/10 |

Sample assigned an asterisk (*) has the recommended dose of a few drops, and the calculation is based on three drops per portion.
IA = Inactive. B = bioactive sample but below EC_20_.

Figure SI-1

**
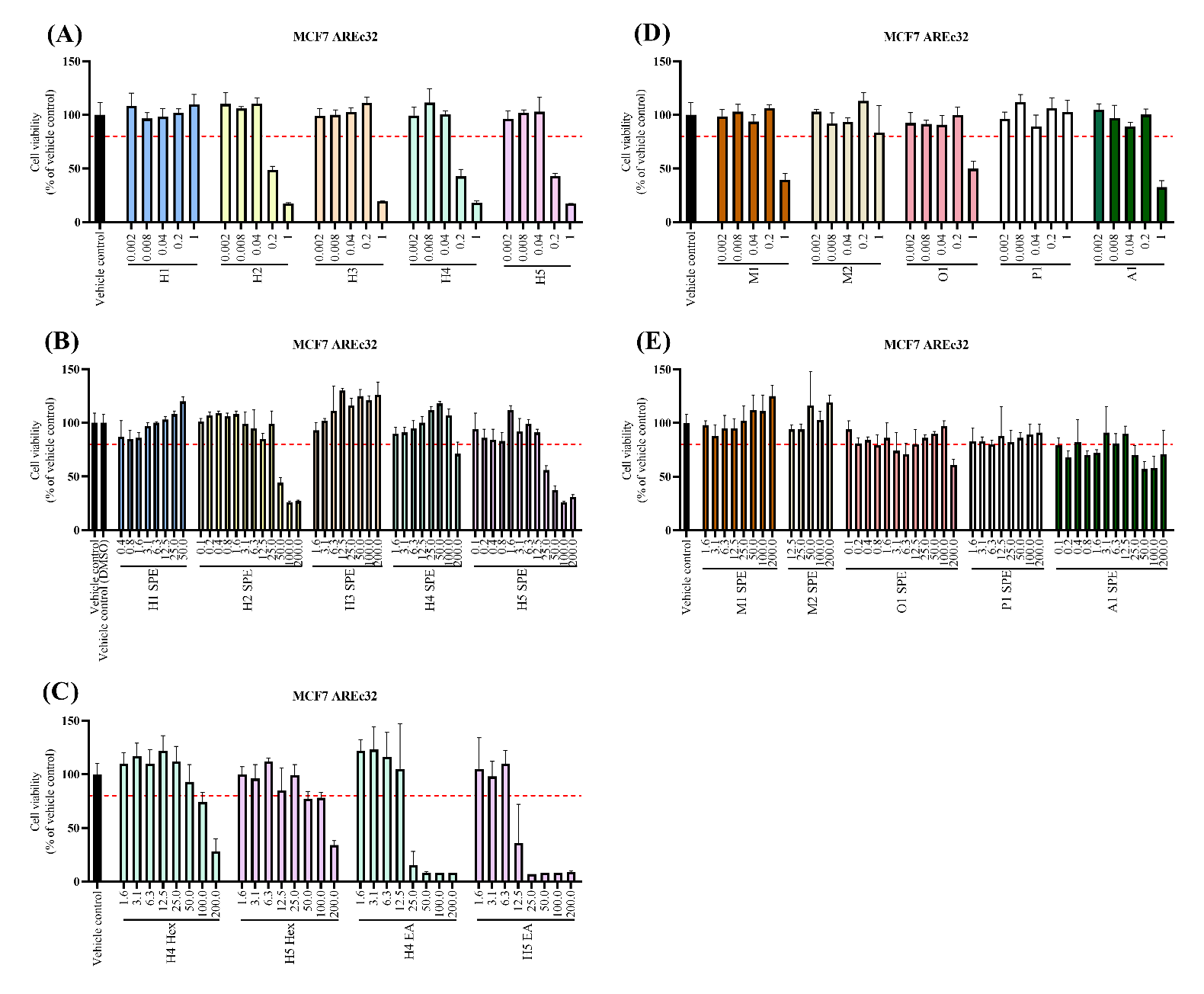
**

**Fig. SI-1:** Cell viability response (% of control) upon 24 h exposure of MCF7 AREc32 cells to liquid smoke flavourings: non-extracted (A, D), SPE extracted (B, E) and LLE extracted (C). Concentration on the x-axis is expressed as μL of liquid smoke flavouring/mL cell culture medium. Data is presented as mean ± SD (n = 4) and the dotted line represents the cut‑off, which was set to 80% cell viability.

Fig. SI-2

**
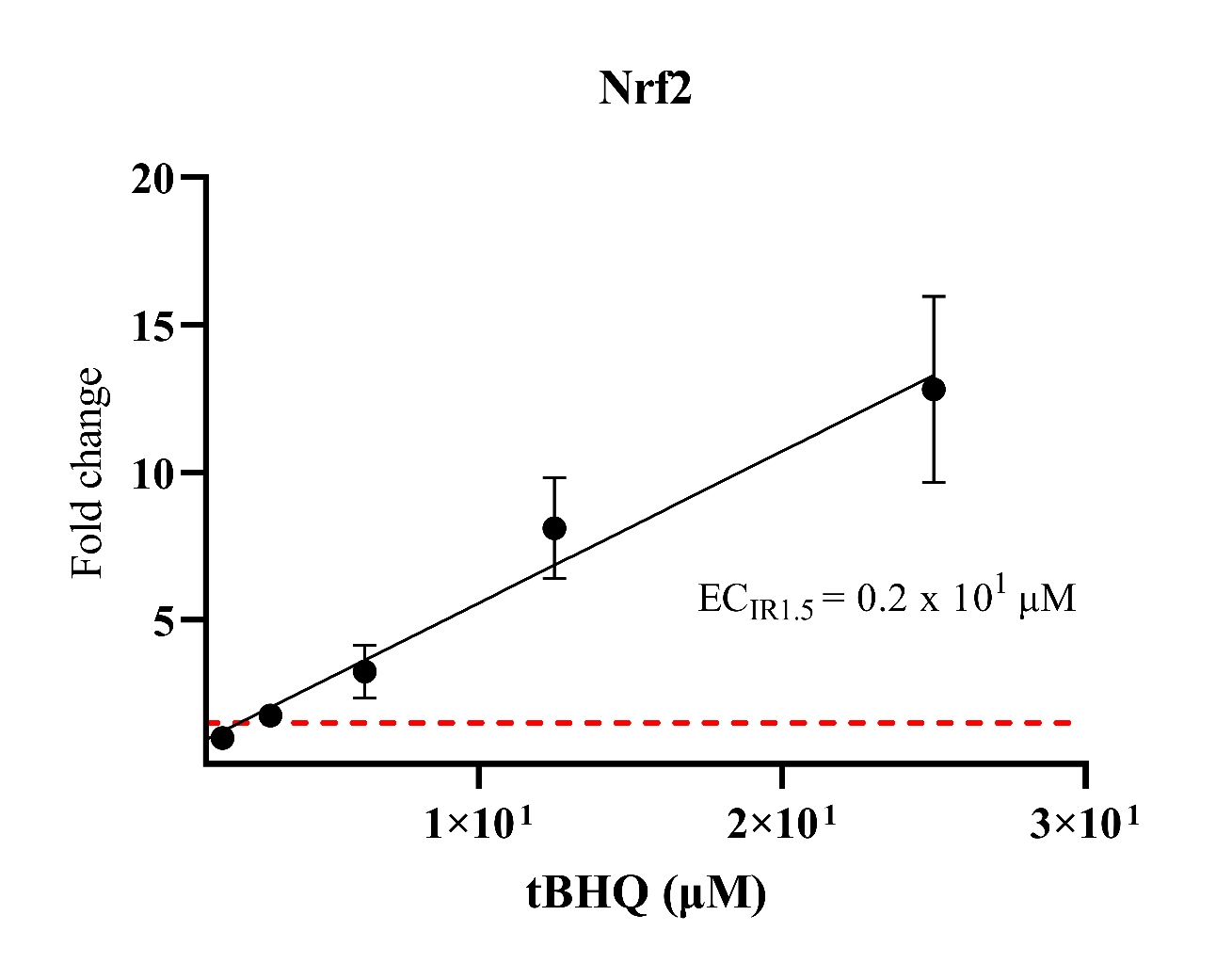
**

**Fig. SI-2:** Oxidative stress response (fold change of Nrf2 compared to control) upon 24 h exposure of MCF7 ARE32 cells to the reference compound tBHQ, used as a standard for Nrf2 oxidative stress response. Concentrations tested ranged were 8 x 10^-1^ – 2.5 x 10^1^ μM. Data illustrates mean ± SD (n = 8) and the dotted line represent the cut-off of 1.5, defined as the cut-off limit of bioactivity.

Fig. SI-3


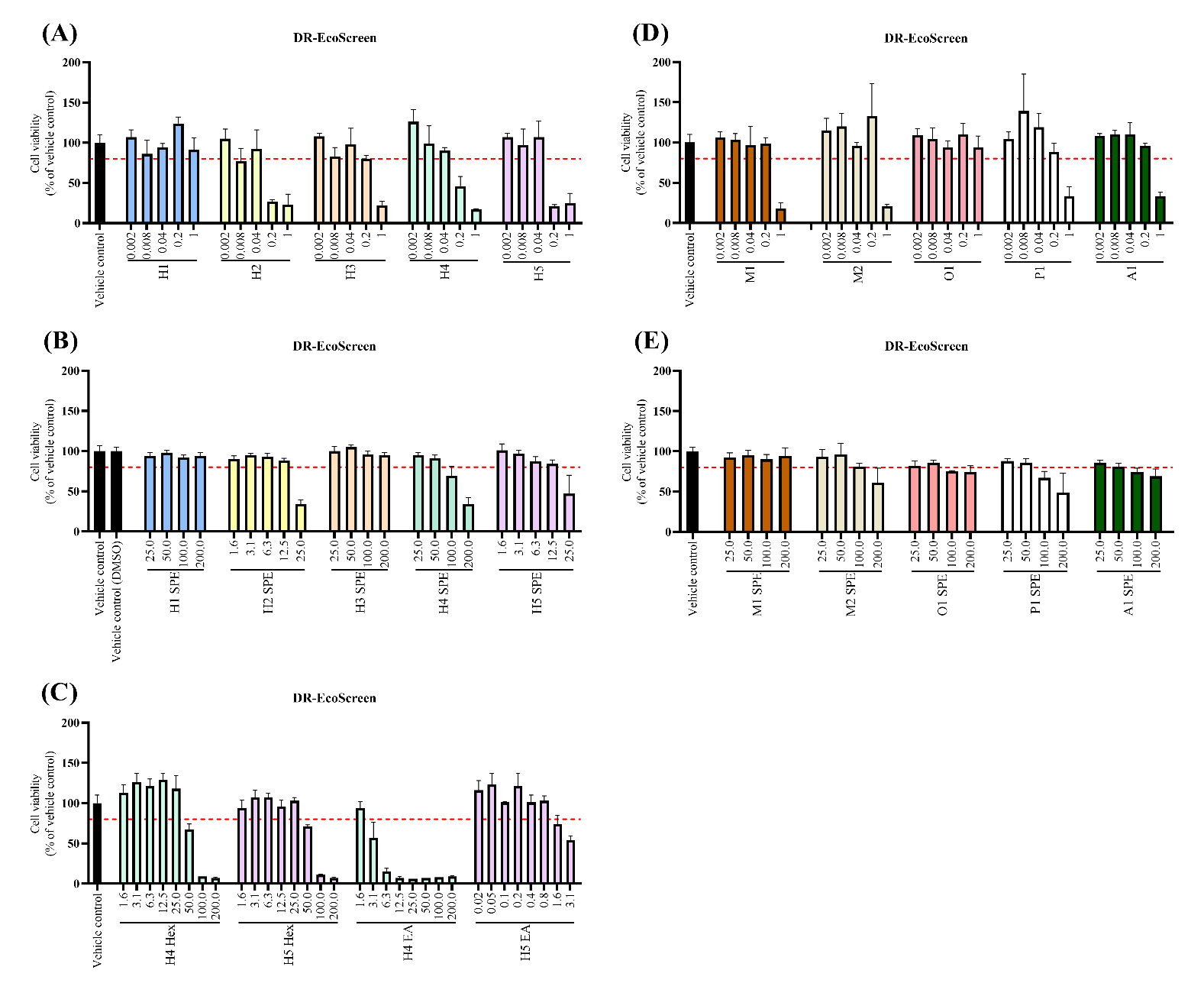


**Fig. SI-3:** Cell viability response (% of control) upon 24 h exposure of DR‑EcoScreen cells to liquid smoke flavourings: non-extracted (A, D), SPE extracted (B, E) and LLE extracted (C). Concentration on the x-axis is expressed as μL of liquid smoke flavouring/mL cell culture medium. Data is presented as mean ± SD (n = 4) and the dotted line represents the cut‑off, which was set to 80% cell viability.

SI-4


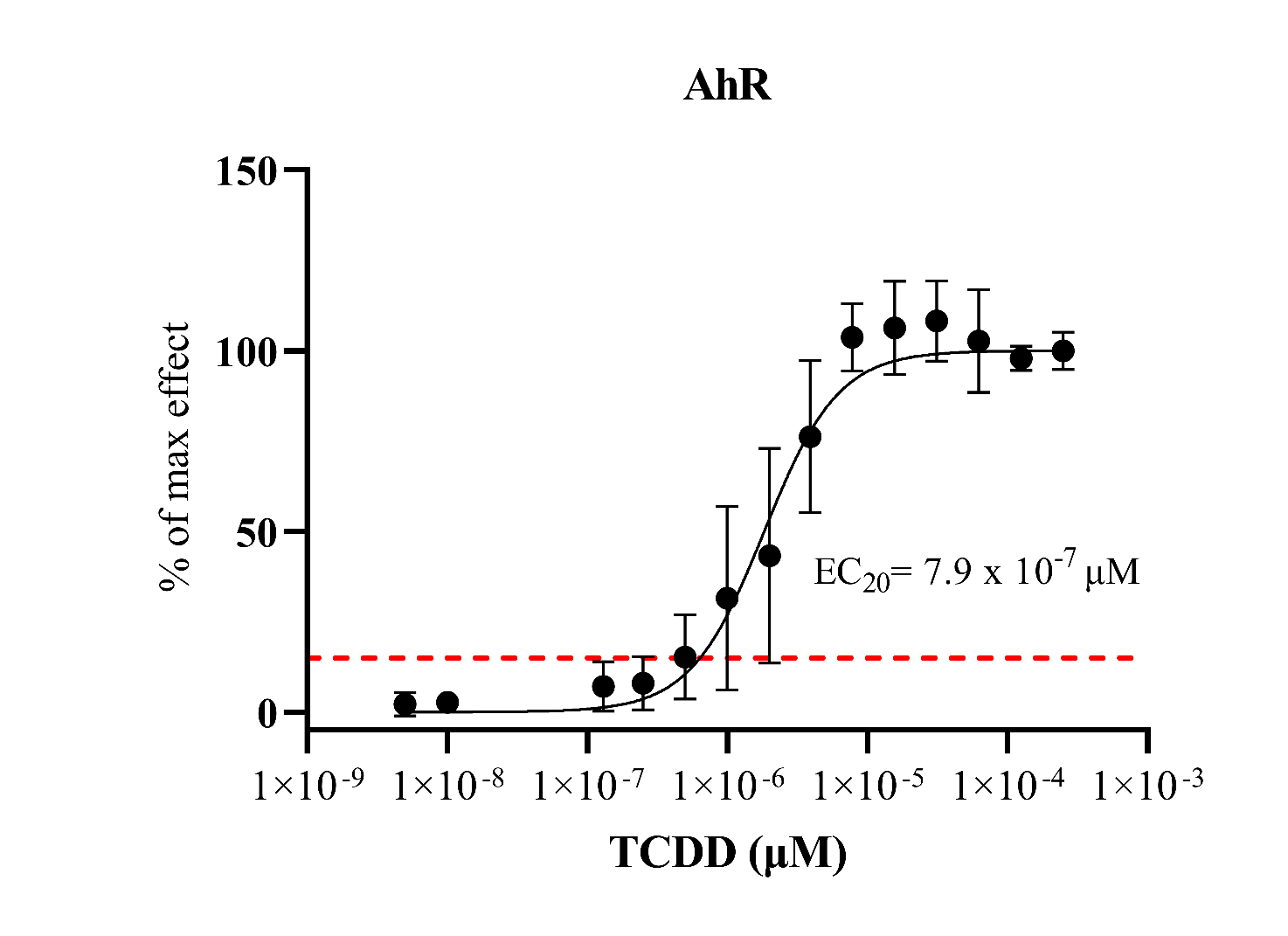


**Fig. SI-4:** AhR activity (% of max effect) after 24 h exposure of DR-EcoScreen cells to the reference compound TCDD, used as a standard for AhR agonistic response. Concentrations tested ranged from 1 x 10^-8^ – 3 x 10^-4^ μM. Data illustrates mean ± SD (n = 8), and the dotted line represent the % max effect of 15, defined as the cut-off limit of bioactivity.

Fig. SI-5


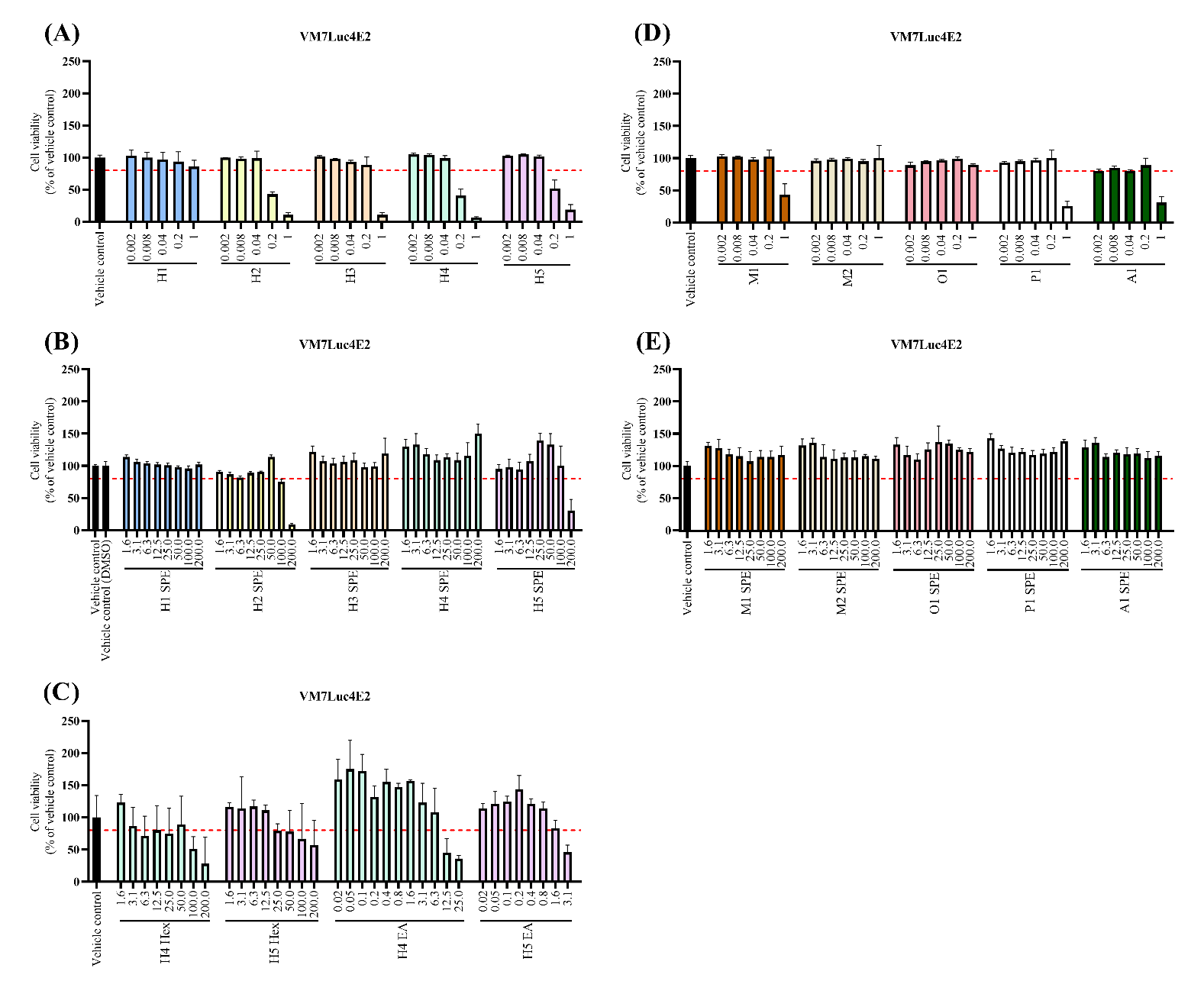


**Fig. SI-5:** Cell viability response (% of control) upon 24 h exposure of VM7Luc4E2 cells to liquid smoke flavourings: non-extracted (A, D), SPE extracted (B, E) and LLE extracted (C). Concentration on the x-axis is expressed as μL of liquid smoke flavouring/mL cell culture medium. The number of technical repeats (n) were 12 for the negative control DMSO for LLE. Data is presented as mean ± SD (n = 4) and the dotted line represents the cut-off, which was set to 80% cell viability.

Fig. SI-6


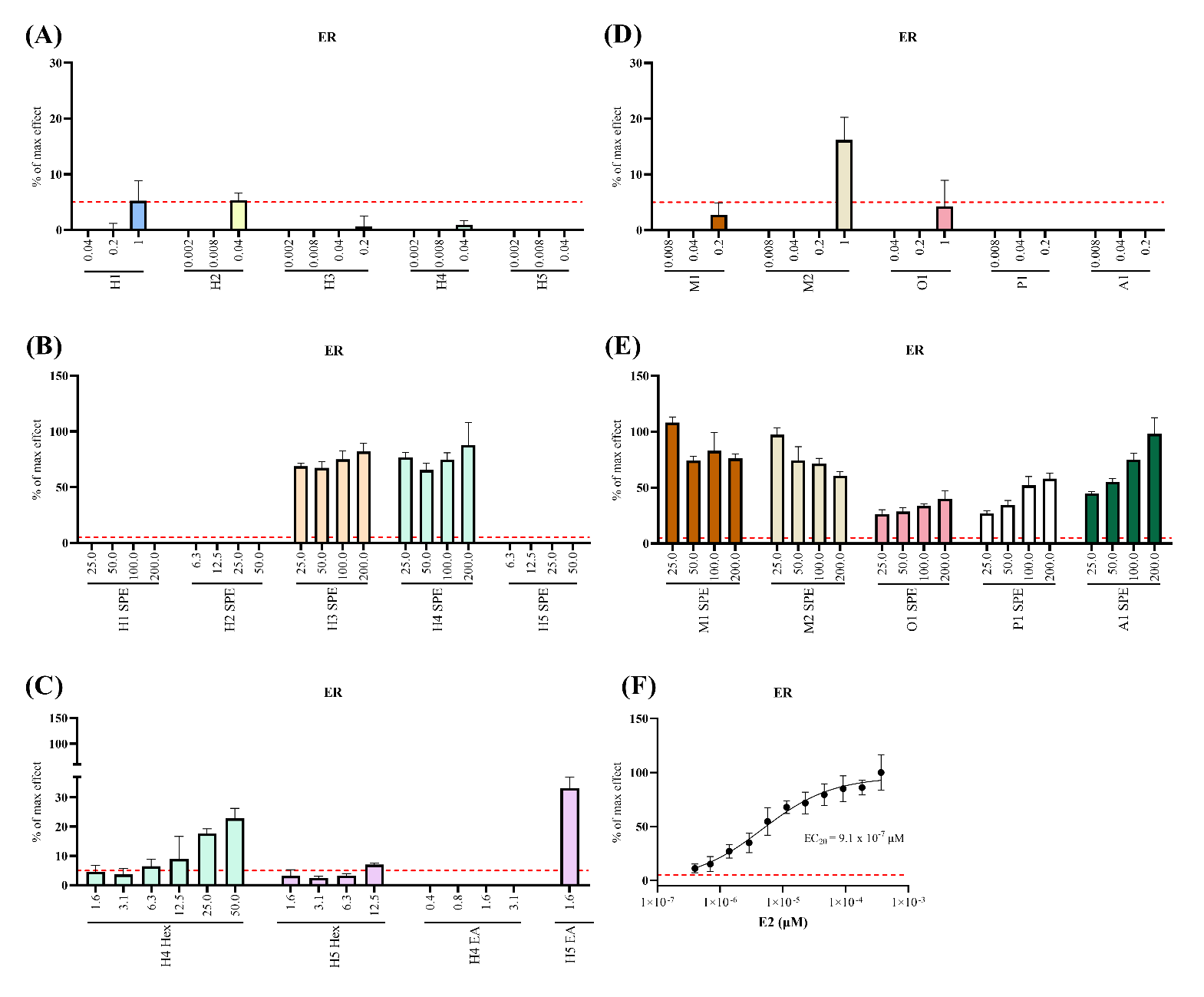


**Fig. SI-6:** ER agonistic response (% of max effect) upon 24 h exposure of VM7Luc4E2 cells to liquid smoke flavourings: non-extracted (A, D), SPE extracted (B, E) and LLE extracted (C). Concentrations on the x-axis are expressed as μL liquid smoke flavouring/mL cell culture medium. Data is illustrated as mean ± SD (n = 4), and the dotted line represents the % assay max of 5, defined as the cut-off limit of bioactivity. Figure (F) illustrates the reference compound E2, used as a standard for ER agonistic response. Concentrations tested ranged from 4 x 10^-7^ – 4 x 10^-4^ μM (n = 8).

Fig. SI-7


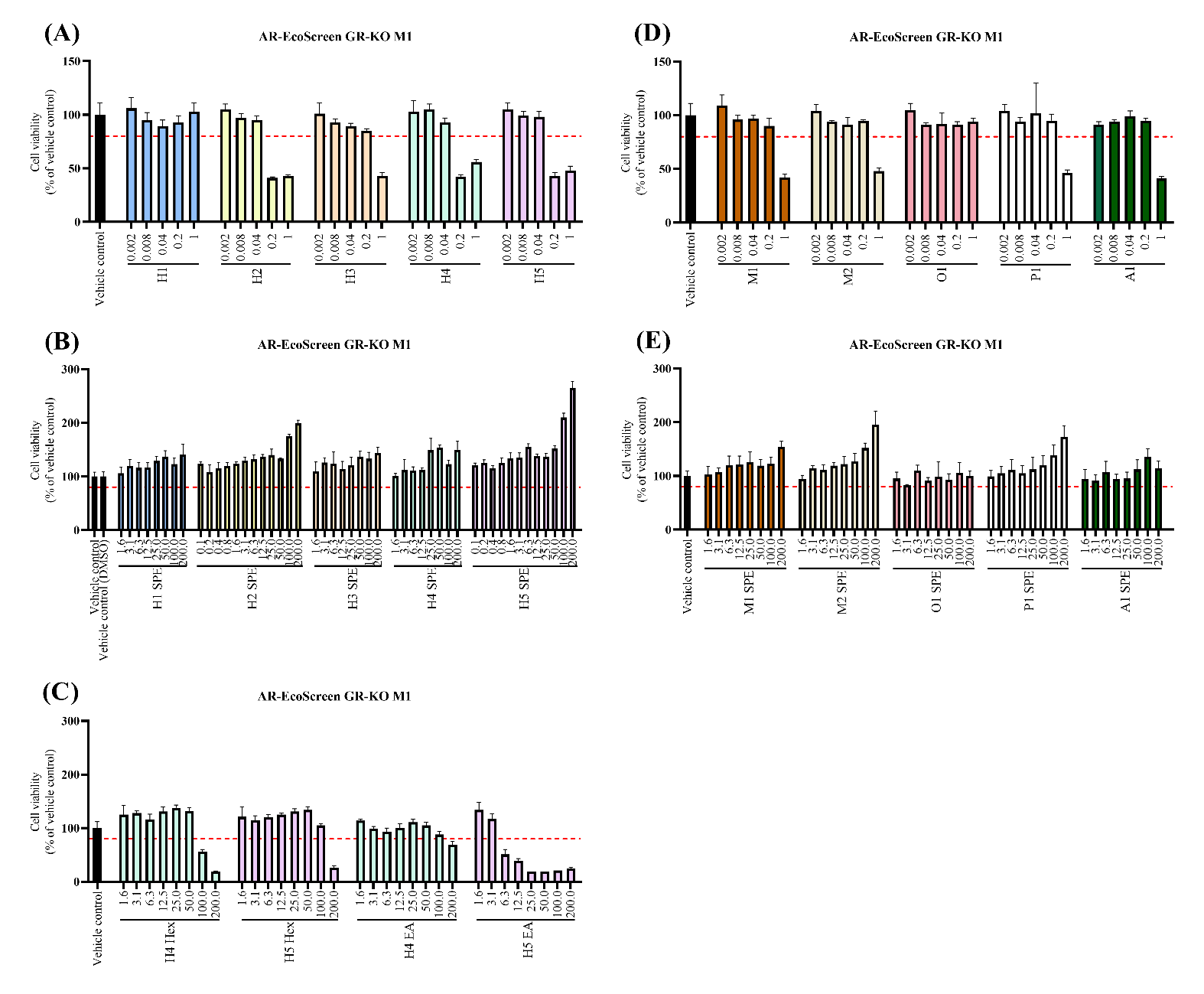


**Fig. SI-7:** Cell viability response (% of control) upon 24 h exposure of AR-EcoScreen GR‑KO M1 cells to liquid smoke flavourings: non-extracted (A, D), SPE extracted (B, E) and LLE extracted (C). Concentration on the x-axis is expressed as μL of liquid smoke flavouring/mL cell culture medium. The number of technical repeats were 8 for the negative control DMSO for liquid-liquid extracted control, and 4 for the samples and cell culture medium control. Data is presented as mean ± SD and the dotted line represents the cut-off, which was set to 80% cell viability.

Fig. SI-8

**
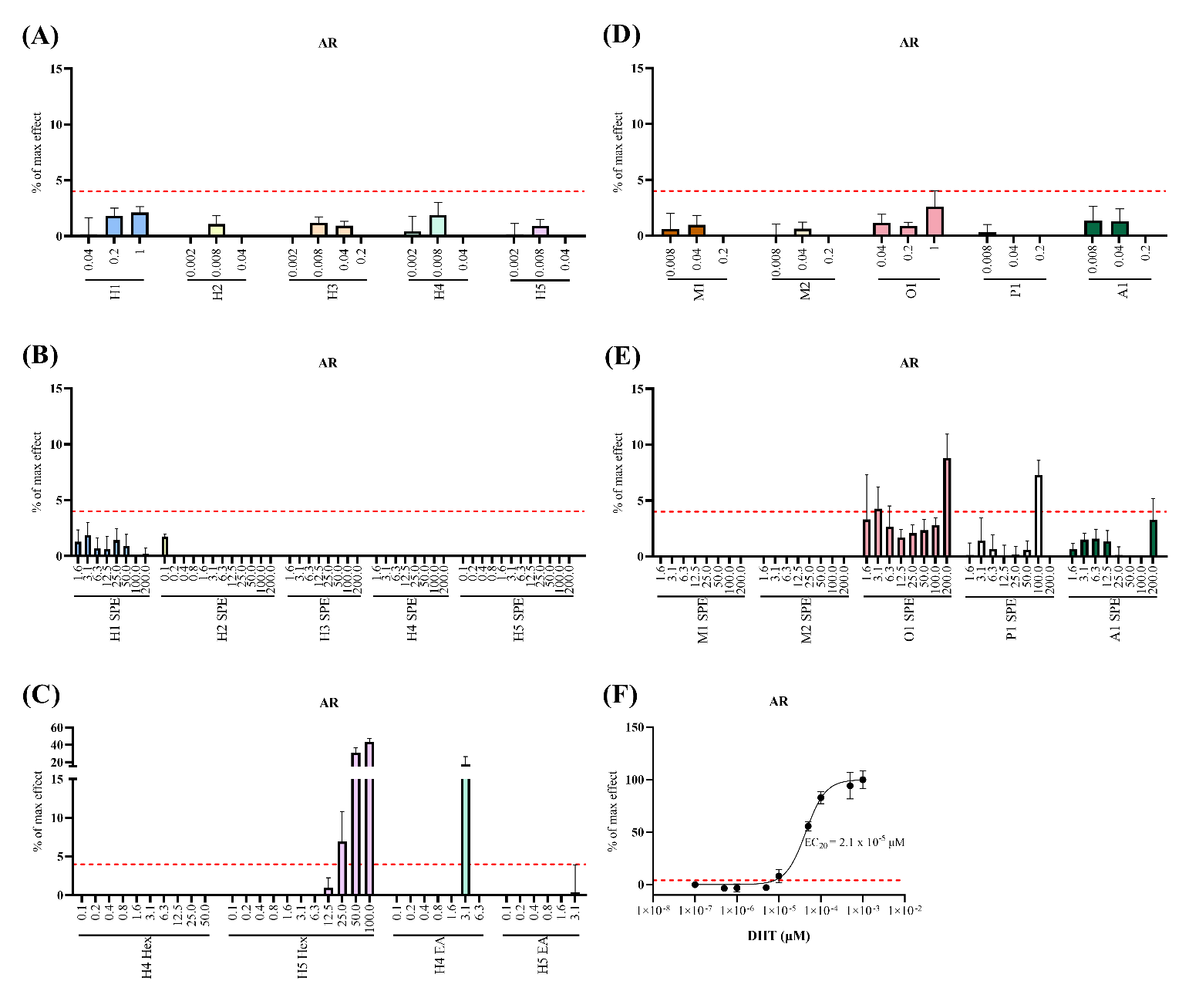
**

**Fig. SI-8:** AR agonistic response (% of max effect) upon 24 h exposure of AR-EcoScreen GR-KO M1 cells to liquid smoke flavourings: non-extracted (A, D), SPE extracted (B, E) and LLE extracted (C). Concentrations on the x-axis are expressed as μL liquid smoke flavouring/mL cell culture medium. Data is illustrated as mean ± SD (n = 4) and the dotted line represents the % assay max of 4, defined as the cut-off limit of bioactivity. Figure (F) illustrates the reference compound DHT, used as a standard for AR agonistic response. Concentrations tested ranged from 1 x 10^-9^ – 1 x 10^-3^ μM (n = 8).

Fig. SI-9


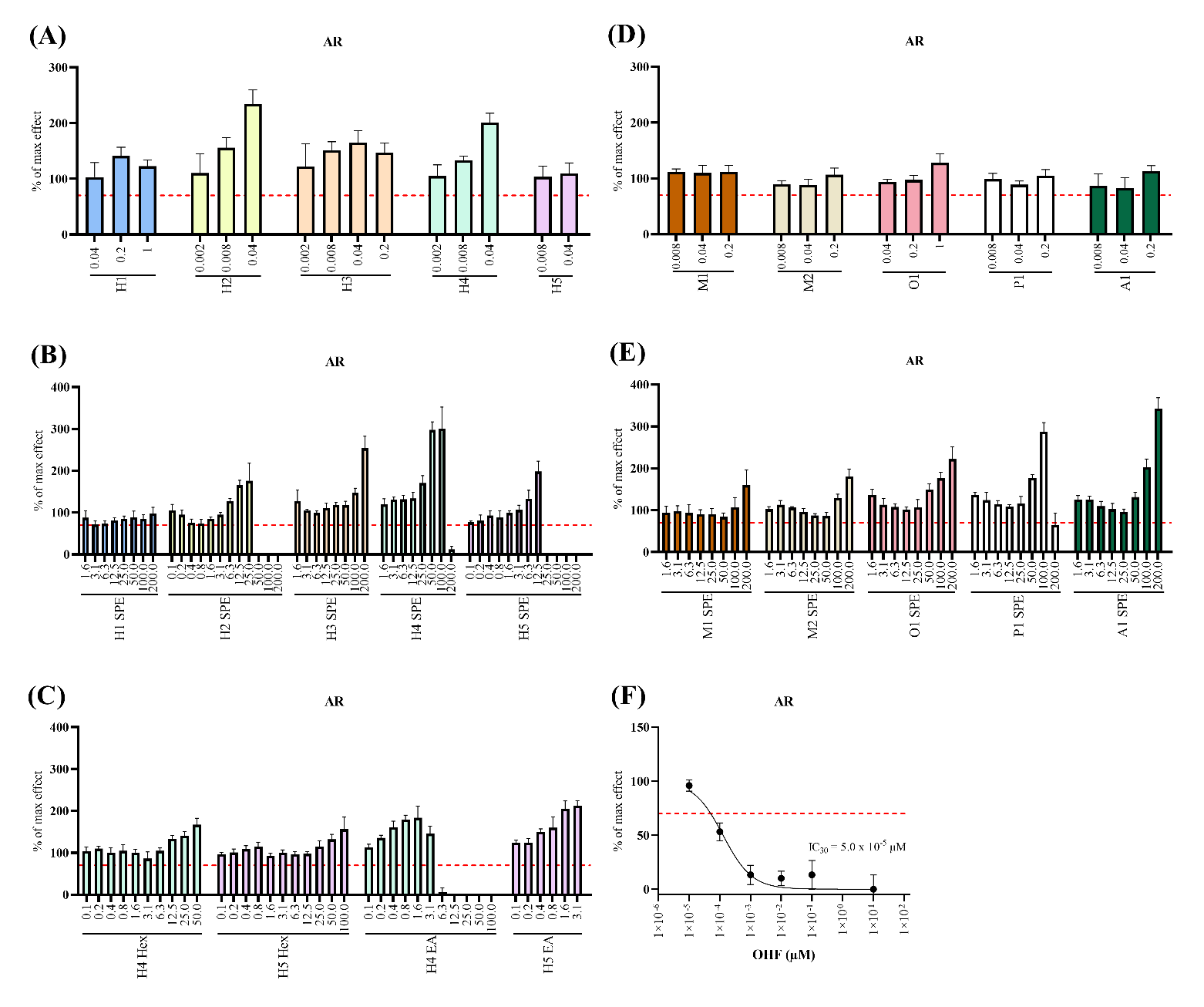


**Fig. SI-9:** AR antagonistic response (% of max effect) upon 24 h exposure of AR-EcoScreen GR-KO M1 cells to liquid smoke flavourings: non-extracted (A, D), SPE extracted (B, E) and LLE extracted (C). Concentrations on the x-axis is expressed as μL of liquid smoke product/mL cell culture medium. Data is illustrated as mean ± SD (n = 4) and the dotted line represent the % assay max of 70, defined as the cut-off limit of bioactivity. Figure (F) illustrates the reference compound OHF, used as a standard for AR antagonistic response. Concentrations tested ranged from 1 x 10^-5^ – 1 x 10^1^ μM (n = 8).
